# Use of Human Induced Pluripotent Stem Cell-Derived Cardiomyocytes to Predict the Cardiotoxicity Potential of Next Generation Nicotine Products

**DOI:** 10.3389/ftox.2022.747508

**Published:** 2022-02-16

**Authors:** Liam Simms, Fan Yu, Jessica Palmer, Kathryn Rudd, Edgar Trelles Sticken, Roman Wieczorek, Fiona Chapman, Lukasz Czekala, Matthew Stevenson, Grant O’Connell

**Affiliations:** ^1^ Imperial Brands PLC, Bristol, United Kingdom; ^2^ Stemina Biomarker Discovery Inc., Madison, WI, United States; ^3^ Reemtsma Cigarettenfabriken GmbH, Hamburg, Germany

**Keywords:** cigarette, NGP, nicotine, cardiotoxicity, hiPSC-CM, *in vitro*, NAMs

## Abstract

Combustible cigarette smoking is an established risk factor for cardiovascular disease. By contrast, the cardiotoxicity potential of non-combustible next generation nicotine products (NGPs), which includes heated tobacco products (HTPs) and electronic vaping products (EVPs), and how this compares relative to combustible cigarettes is currently an area of scientific exploration. As such, there is a need for a rapid screening assay to assess this endpoint. The Cardio *quick*Predict is a metabolomics biomarker-based assay that uses human induced pluripotent stem cell-derived cardiomyocytes (hiPSC-CM) to screen for potential structural and functional cardiac toxicants based on the changes of four metabolites, lactic acid, arachidonic acid, thymidine, and 2′-deoxycytidine. The study aims were to investigate the cardiotoxicity potential of NGPs compared to cigarettes, in addition to nicotine. To accomplish this, hiPSC-CM were exposed to smoke or aerosol bubbled PBS samples: reference cigarette (1R6F); three variants of HTP; and three EVP variants. The 1R6F bPBS was the most active, having cardiotoxic potential at 0.3–0.6% bPBS (0.4–0.9 μg/mL nicotine), followed by HTP, which displayed cardiotoxic potential at a 10 times higher concentration, 3.3% bPBS (4.1 μg/mL nicotine). Both 1R6F and HTP bPBS (at 10-fold higher concentration than 1R6F) affected all four predictive metabolites, whereas none of the EVP bPBS samples were active in the assay up to the maximal concentration tested (10% bPBS). Nicotine tested on its own was predicted to have cardiotoxic potential at concentrations greater than 80 μg/mL, which is higher than expected physiological levels associated with combustible cigarette smoking. The application of this rapid screening assay to NGP research and the associated findings adds to the weight-of-evidence indicating that NGPs have a tobacco harm reduction potential when compared to combustible cigarettes. Additionally, this technique was shown to be sensitive and robust for the assessment of different NGPs and may be considered as part of a larger overall scientific framework for NGP assessments.

## 1 Introduction

Cigarette smoking is a cause of serious diseases in smokers and is an established risk factor for cardiovascular disease (Messner and Bernhard, 2014). Cigarette smoke is a complex mixture comprised of both gas and particle phases containing around 7,000 chemical constituents, (Smith et al., 2001; Rodgman and Perfetti, 2008; Rodgman, 2014). Around one hundred of these chemicals have been identified as harmful and potentially harmful constituents (HPHCs) by public health bodies (Burns et al., 2008; FDA, 2012). Certain HPHCs have been classified as having cardiotoxic activities, including: carbon monoxide, acrolein, arsenic, benz [a]anthracene, benzene, benzo [b]fluoranthene, benzo [k]fluoranthene, chrysene, cobalt, hydrogen cyanide, lead, phenol, and propionaldehyde (FDA, 2012). Non-combustible nicotine products, which deliver nicotine but with fewer and lower levels of toxicants compared to cigarette smoke, represent a promising tobacco harm reduction (THR) tool for adult smokers who are unwilling or uninterested to quit smoking and who would otherwise continue to smoke. Collectively these are known as Next Generation Products (NGPs) and encompass many non-combustible tobacco as well as tobacco-free products, including heated tobacco products (HTPs) and electronic vapour products (EVPs). Whereas EVPs aerosolise a nicotine-containing liquid (typically with propylene glycol and vegetable glycerine carriers) to create an inhalable aerosol, HTPs electronically heat a portion of tobacco at temperatures well below the level necessary to burn tobacco to generate the inhalable aerosol. By eliminating tobacco combustion, NGP aerosols have been shown to have a relatively simple chemical composition with fewer and substantially reduced levels of HPHCs and other chemicals when compared to tobacco smoke (Schaller et al., 2016b; Jaccard et al., 2017; Eaton et al., 2018; Forster et al., 2018; Bentley et al., 2020; Rudd et al., 2020).

Cigarette smoking, and associated HPHCs, are major causes of cardiovascular disorders including coronary heart disease, stroke, aortic aneurysm, and peripheral artery disease (Benowitz and Burbank, 2016). The risk is seen both as an increased risk of acute thrombosis of narrowed vessels and as an increased degree of atherosclerosis in the blood vessels involved and damage to the myocardium. Cigarette smoke -induced cardiac damage is divided into two major and interchangeable mechanisms consisting of 1) direct adverse effects on the myocardium causing smoking cardiomyopathy due to oxidative stress, inflammation metabolic impairment and cell death or 2) *via* indirect effects on the myocardium by causing comorbidities such as the development of atherosclerosis and hypertension that eventually leads to damage and remodelling of the heart (Kaplan et al., 2017). The cardiovascular risks attributable to cigarette smoking increase with the number of cigarettes smoked and with the duration of smoking (US Surgeon General, 2010) and there are a number of mechanisms involved in the development of associated cardiovascular disease pathologies. These include, inflammation, endothelial dysfunction, lipid abnormalities, such as oxidation of low-density lipoprotein (LDL), and platelet activation (Burke and FitzGerald, 2003); all of these are associated with atherogenic and atherosclerotic progression, in addition to the key role of oxidative stress (Marchio et al., 2019).

Cigarette smoke is known to induce cellular oxidative stress, which is in fact reported to be a driving factor in many smoking related diseases (Laniado-Laborin 2009; Kamceva et al., 2016). Extensive research conducted over the last 30 years has revealed the mechanism by which continued oxidative stress can lead to chronic inflammation, ultimately leading to numerous chronic diseases including diabetes, cancer, cardiovascular, neurological, and pulmonary diseases (Reuter et al., 2010). Oxidative stress has been shown to activate various transcription factors including NF-κB, AP-1, p53, HIF-1α, PPAR-γ, β-catenin/Wnt and Nrf2, which leads to changes in the expression of multiple genes, including growth factors, inflammatory cytokines, chemokines, molecules regulating cell cycle, and anti-inflammatory molecules (Reuter et al., 2010). Delivery of free radicals and oxidants present in both the gas and particulate phases of cigarette smoke, as well as endogenously produced oxidants and radicals (resulting from the smoke chemical-induced alteration in the cellular redox system), cause a pro-oxidative environment. This general shift is likely to contribute to lipid oxidation and to a general increase in oxidative modification (and inactivation) of biomolecules (Messner and Bernhard, 2014). Morrow et al. (1995) reported the increased presence of lipid peroxidation products in the serum of smokers due to the stable aldehydes in cigarette smoke increasing reactive oxygen species production by the activation of NADPH oxidases (Messner and Bernhard, 2014).

Mitochondrial dysfunction caused by cigarette smoke is involved in the pathology of respiratory diseases caused by oxidative stress and reducing the levels of harmful and potentially harmful components, by heating instead of burning tobacco, can further reduce mitochondrial changes that contribute to oxidative stress and cell damage (Malinski et al., 2018). However, there is some evidence that NGPs may demonstrate some association with oxidative stress and also cardiovascular disease. NASEM (2018), in their review of e-cigarettes concluded that “There is substantial evidence that components of e-cigarette aerosols can promote formation of reactive oxygen species/oxidative stress. Although this supports the biological plausibility of tissue injury and disease from long-term exposure to e-cigarette aerosols, generation of reactive oxygen species and oxidative stress induction is generally lower from e-cigarettes than from combustible tobacco cigarette smoke.” Buchanan et al. (2020) undertook a review of the clinical and pre-clinical studies conducted on e-cigarettes and concluded that the studies presented in their review “have shown that e-cigarettes can induce negative cardiovascular effects through various mechanisms such as oxidative stress, inflammation, DNA damage, arterial stiffness, and altered haemodynamics, and platelet activation.” Furthermore, recent research suggests that HTP and EVP aerosols can affect the cardiovascular system, albeit it at exposures many magnitudes higher than combustible cigarette cardiotoxic exposures (Abouassali et al., 2021; Qasim et al., 2017; Znyk et al., 2021). The role of NGPs in relation to cardiovascular diseases is however, still a source of debate and controversy (NASEM 2018; Fried and Gardner, 2020; Tsai et al., 2020). Given the reported association between exposure to cigarette smoke and cardiovascular disease risk, and emerging evidence of potential effects of NGPs, there is a need for models to screen for such effects and to determine the potential of NGPs to induce cardiotoxicity. These models would ideally be high throughput and possess human relevance. Gathering mechanistic information on NGP samples relative to combustible cigarette would also help to determine their THR potential and to determine if the novel aerosol mixtures have the potential to induce cardiotoxicity, preferably using a high throughput human cell-based system, for increased human relevance. The implementation of rapid screening assays that can identify functional and structural cardiotoxicity earlier in the drug development pipeline has been shown to have the potential to improve safety and the cost and time required to bring new drugs to market (Palmer et al., 2020). With respect to NGPs, such assays allow for a broad screening assessment of potential toxicity when used as part of a wider overall scientific assessment framework for substantiating the THR potential of products as part of a weight-of-evidence approach. Current *in vitro* testing strategies for cardiotoxicity predominantly focus on the compound impact on individual ion channels. In addition, pre-clinical animal models used to assess cardiotoxicity are limited due to being low throughput, costly and may lack human relevance (Burnett et al., 2021). The Stemina Cardio *quick*Predict™ (Cardio^*qP*^) is a human induced pluripotent stem cell-derived cardiomyocyte (hiPSC-CM)-based assay that predicts a test sample’s cardiotoxicity potential using changes in hiPSC-CM metabolism and cell viability. The assay was developed and commercialized by Stemina Discovery Inc. The assay’s metabolic endpoints are generated using known structural and functional reference toxicants to identify predictive metabolites released in the secretome of the hiPSC-CM. The assay’s prediction model uses four metabolites (lactic acid, 2′-deoxycytidine, thymidine, and arachidonic acid) and cell viability to predict the concentration at which a test sample shows cardiotoxicity potential (cTP). These metabolites were found to be the most responsive when hiPSC-CM were exposed to known cardiotoxic drugs (Palmer et al., 2020). The reported accuracy, sensitivity, and specificity of the Cardio^*qP*^ assay are 86%, and 90%, respectively (Palmer et al., 2020). hiPSC-CM expresses most of the human cardiac ion channels and sarcomeric proteins, some of which contribute to action potential generation and are regulated by adrenergic stimulation (Huo et al., 2017; Zhao et al., 2018; Burnett et al., 2021). This assay adds significantly to other cardiac assays available when used in isolation, or as part of a combined approach, and can be used to screen test articles for potential cardiotoxicity.

To allow for the *in vitro* exposure of cigarette smoke and NGP aerosols, a number of trapping methodologies have been employed (Smart and Phillips, 2021). The bubbling of whole smoke or aerosol in phosphate buffered saline (bPBS) is a commonly used method and allows for the direct addition of bPBS into an *in vitro* cell system (Johnson et al., 2009). The bubbling procedure largely captures the water-soluble gaseous fraction of the smoke or aerosol, making this an alternative delivery mechanism to the historically used filter trapped total particulate matter or aerosol collected mass only. Work by Buratto et al., has shown that a number of carbonyls can be successfully trapped and quantified within bPBS from both combustible cigarettes and HTPs (Buratto et al., 2018).

This study aimed to screen the cardiotoxic potential of six NGP aerosols (from three HTP variants and three EVP variants) and reference cigarette smoke (1R6F), trapped directly in PBS, and then added to the cell culture medium of hiPSC-CMs. For each test sample, the concentration predicted to have cardiotoxic potential was determined using the Cardio*qP* prediction model. Nicotine was also tested in isolation to assess its effects in this system. To our knowledge, this study provides the first published assessment of the cardiotoxicity potential for a variety of NGP aerosols and cigarette smoke using the Cardio^*qP*^ assay.

## 2 Materials and Methods

### 2.1 Test Samples

The test samples evaluated in this study with the Cardio^*qP*^ assay are described below in Table 1. The test sample were evaluated in three separate batches in 2019 (study 1: 1R6F S1 and cHTP, EVP-FB, EVP-0 and EVP-NS; study 2: 1R6F S2, pHTP1, pHTP2; study 3: nicotine). The bPBS samples for the studies were analysed within 3–4 months of generation.

**TABLE 1:** Test samples evaluated in the Cardio^qP^ assay.

Study	Sample	Product category	Coding	Manufacturer
1, 2	1R6F reference cigarettes	Cigarette	1R6F S1^a^	University of Kentucky
			1R6F S2^b^	
1	IQOS with Amber HEETS	HTP	cHTP	Philip Morris International
2	Prototype HTP	HTP	pHTP1^c^	Imperial Brands
2	Prototype HTP	HTP	pHTP2^d^	Imperial Brands
1	*my*blu with tobacco flavour, 0% nicotine^e^	EVP	EVP-0	Imperial Brands
1	*my*blu with tobacco flavour, 1.6% nicotine (freebase)^e^	EVP	EVP-FB	Imperial Brands
1	*my*blu with tobacco flavour, 1.6% nicotine salt (nicotine lactate)^e^	EVP	EVP-NS	Imperial Brands
3	Nicotine	—	—	Sigma-Aldrich

a 1R6F S1 1R6F reference cigarette used in Study 1

b 1R6F S2 1R6F reference cigarette used in Study 2

c pHTP1 Imperial Brands prototype HTP with tobacco variant consumables.

d pHTP2 Imperial Brands prototype HTP with menthol variant consumables.

e All *my*blu e-liquids were custom made by Nerudia Ltd., a subsidiary of Imperial Brands, for research purposes only. The nicotine was derived from tobacco.

Prior to smoke/aerosol generation, the 1R6F reference cigarette (batch #V062X53D1), and the respective HTP tobacco consumable sticks were stored in an airtight container at 4°C until use. The reference cigarettes and HTP sticks were allowed to come to room temperature for 15 min before opening and conditioned for at least 48 h in a standard humidified chamber following the International Organization for Standardisation (ISO) 3402 Guideline (International Organization for Standardization, 1999). Additional information of the device used in this study is proved in the Supplementary Material.

### 2.2 Smoke and Aerosol Extract Generation

Fresh whole aerosol and smoke was trapped in phosphate-buffered saline (bPBS) using the VITROCELL VC 10 S-TYPE smoke robot (VITROCELL Systems GmbH, Waldkirch, Germany), connected to glass impingers containing 30 mL PBS in total. The smoke from the 1R6F reference cigarette and aerosols from HTPs and EVPs were generated as detailed in Table 2. For the 1R6F reference cigarette a total of 56 puffs (8 puffs per stick, 7 cigarette sticks) per 30 mL (1.87 puffs per mL) PBS was generated. For all the NGPs, a total of 120 puffs (for pHTPs: 8 puffs/stick, 15 sticks; for cHTP: 10 puffs/stick, 12 sticks; for EVPs: 60 puffs per device, 2 devices) per 30 mL (4 puffs per mL) PBS were generated. Smoke or aerosol extracts were prepared by bubbling the sample generated smoke or aerosol into 3 in-line impingers, each containing 10 ml PBS. The three 10 ml samples were then combined to make a total stock solution of 30 mL prior to being aliquoted, rapidly frozen, and stored at −70°C, Samples were shipped on dry ice to Stemina, where the biological assay was carried out. The 30 mL PBS stocks contained 56 puffs for 1R6F and 120 Puffs for the NGPs to reduce the length of time the samples were collected in. All samples were collected over half an hour to try to minimise losses of any volatile constituents, that may be seen with an extended collection time (hours) for higher puff numbers per mL of PBS. The 56 Puffs for 1R6F was based on the cytotoxicity seen with higher puff numbers. Nicotine for the nicotine sample was simply diluted in the culture medium (containing 0.1% DMSO) at a concentration of 1 mg/ml).

**TABLE 2:** Smoke/aerosol generation standardized parameters.

Product	Smoking regime	Total puff number	Puffs per mL PBS
1R6F Reference cigarette	ISO 20779:2018 intense smoking regime (International Organization for Standardization, 2018b)	56	1.87
	*(bell-shaped puff profile, 55 mL puff volume, 2 sec duration, 30 sec interval with 100% ventilation blocking)*		
HTPs	Modified ISO 20779:2018 (International Organization for Standardization, 2018b)	120	4
	*(bell-shaped puff profile, 55 mL puff volume, 2 sec duration, 30 sec interval, no ventilation blocking)*		
EVPs	ISO 20768:2018 (International Organization for Standardization, 2018a)	120	4
	*(square-wave puff profile, 55 mL puff volume, 3 sec duration and a 30 sec interval, no ventilation blocking)*		

The smoke/aerosol generation was performed using the standardized methods adopted by ISO to ensure repeatability of sample generation. All samples used a 55 mL puff volume collected over 2 or 3 sec. there was a 30 sec interval between puffing. The bell and square wave puff profile relates to the the smolder phase between puffing from cigarettes (bell shaped), square wave due to the either on or off nature of the heating elements for the NGPs. sec = second

### 2.3 Characterisation of bPBS

Nicotine and eight selected carbonyls, based on the list described by Buratto et al. (2018), were quantified within the aerosol and smoke bPBS samples using the previously described methodology (Simms et al., 2020; Czekala et al., 2021). In brief, nicotine was quantified using liquid chromatography with tandem mass spectrometry (LC-MS/MS) with an AB Sciex API 6500 QTRAP (SCIEX, Framingham, MA, United States). Carbonyl detection methodology in bPBS was adapted from ISO 21160:2018 (ISO, 2018) methodology for carbonyl detection. Carbonyl-DNPH derivates, were quantified using high-performance liquid chromatography with a diode-array detector (HPLC-DAD, Agilent Technologies 1100 Series, Agilent Technologies, Santa Clara, CA, United States).

### 2.4 hiPSC-CM Culture and Treatment

Cryopreserved hiPSC-CM (iCell^®^ Cardiomyocytes) were obtained from FUJIFILM Cellular Dynamics, Inc. (FCDI, Madison, WI, United States) and cultured as previously described (Palmer et al., 2020). Cardio^*qP*^ has not undergone a formal validation study but has been extensively tested in over 80 compounds known to cause a wide range of cardiotoxic effects. Additionally the reproducibility of the Cardio^*qP*^ biomarkers and prediction model was evaluated during assay development (Palmer et al., 2020). Sixty-six drugs were evaluated a minimum of three times by multiple scientists with multiple lots of hiPSC-CM and reagents over the course of 3 years. These studies demonstrated that the drugs altered the metabolism of the same biomarkers at similar concentrations, indicating that both the biomarker response and the concentration where cardiotoxicity potential was observed were reproducible over multiple replicates (Palmer et al., 2020).

HiPSC-CM were exposed to 16 concentrations of nicotine (Sigma-Aldrich, St. Louis, MO, United States) ranging from 0.01 to 1,000 μg/ml. For smoke and aerosol bPBS testing, exposures were based on the bPBS concentration, which was used to calculate nicotine exposures during data analysis. Cells were exposed to eight concentrations of each test article, ranging from 0.053–3% with quarter-log dilutions (1R6F S1 or S2 bPBS) or 0.18–10% with quarter-log dilutions (all NGP bPBS). Test articles were diluted in iCell Cardiomyocytes Maintenance Medium containing 0.1% DMSO such that the final concentration of DMSO was 0.1% in all treatments.

Test article exposure began 4 days after plating. HiPSC-CM were exposed to test article for 72 h, with media ± test article replacement every 24 h. The spent media from the last 24-h treatment period was collected for analysis of metabolites. Cell viability was assessed after sample collection using the CellTiter-Fluor Cell Viability Assay (Promega, Madison, WI, United States). All experimental treatments were carried out in a 96-well plate format. Each test plate included cells exposed to 30 µM verapamil (Toronto Research Chemicals, North York, ON, Canada) as the positive control (*n* = 3 wells), 0.005 µM verapamil as the negative control (*n* = 3 wells), 0.1% DMSO as the reference (vehicle) control (*n* = 3 wells), eight concentrations of two test samples (*n* = 3 wells per concentration), and media controls for each treatment (lacking cells, ± test article). A single biological replicate (or repeat) was performed for each test article.

### 2.5 Sample Preparation

Proteins were precipitated in spent media samples using 60% methanol/40% acetonitrile solution cooled to −20°C containing thymidine-^13^C_10_, ^15^N_2_, 2′-deoxycytidine-^15^N_3_, sodium L-lactate-^13^C_3_ (Cambridge Isotope laboratories, Tewksbury, MA, United States), and arachidonic acid-D_8_ (Sigma-Aldrich). Samples were centrifuged at 2000 × g at 4°C for 10 min to pellet the precipitated proteins and the supernatant was transferred to a new 96-well plate for analysis.

### 2.6 Mass Spectrometry

Ultra-Performance Liquid Chromatography-High Resolution Mass Spectrometry (UPLC-HRMS) data were acquired as described in Palmer et al. (2020) using an Agilent 1290 Infinity LC system interfaced with an Agilent G6530 QTOF high-resolution mass spectrometer (Agilent Technologies). Briefly, an ACQUITY UPLC BEH Amide column (Waters, Milford, MA, United States) maintained at 45°C was used for metabolite separation. A 2 μL volume of sample was injected and data were collected over 7 min using a 6.1-min solvent gradient containing 5 mM ammonium acetate (pH 5.7) in water and 5 mM ammonium acetate (pH 5.7) in 95% acetonitrile.

### 2.7 Quality Control

Two quality control procedures were included to ensure correct plate readout. Firstly, the vehicle control (0.1% DMSO) sample coefficient of variation for the viability relative fluorescent units (RFU) could not exceed 10%. Second, the positive and negative control treatments had to be correctly predicted to ensure that the hiPSC-CM metabolism was within the assay specifications. Quality control data is provided in Supplementary materials (Supplementary Figure S4).

### 2.8 Data Analysis

#### 2.8.1 UPLC-HRMS Data Analysis

The extracted ion chromatogram height for lactic acid, thymidine, arachidonic acid, and 2′-deoxycytidine, and the internal standards (ISTDs) were determined using the Agilent Mass Hunter Quantitative Analysis software, version B.05.00 or newer (Agilent Technologies). The height of each endogenous metabolite was normalized to the corresponding ISTD by dividing the endogenous metabolite height by the corresponding isotopically labelled ISTD height. Relative fold changes were then calculated for each ISTD-normalized metabolite in each sample by dividing the sample response by the median ISTD-normalized response of the reference treatment (0.1% DMSO) samples, producing a reference-normalized value for each metabolite in each sample within a plate of cell culture samples. A Grubbs’ test was then used to identify outlier samples within each treatment and exposure level, and outlier samples were then removed from further analyses.

#### 2.8.2 Viability Data Analysis

To determine the relative fold changes for cell viability, the RFU value for each sample was first background corrected by subtracting the RFU value of the treatment specific media blank from the cell sample RFU. Next, the values were reference-normalized by dividing the background-corrected RFU value of each sample by the average RFU value (background corrected) of the reference treatment.

#### 2.8.3 Prediction Model Calculation

The prediction model uses the reference-normalized response for thymidine and 2′-deoxycytidine and the viability-normalized responses for lactic acid and arachidonic acid. The viability-normalized responses for lactic acid and arachidonic acid were calculated for each sample by dividing the reference-normalized viability value by the reference-normalized lactic acid or arachidonic acid value. The prediction distance for each metabolite was determined by dividing the median metabolite value at a given concentration by its nearest prediction threshold (Table 3). Values outside the prediction thresholds were predicted to be cardiotoxic. The prediction model value (combined prediction distance) at each concentration was the maximum prediction distance of the metabolites used in the model.

**TABLE 3:** Metabolite specific prediction thresholds based on upper lower band beyond which ratios or values are considered to be cardiotoxic.

Metabolite	Lower threshold	Upper threshold
Viability/Lactic Acid	0.325	1.44
Viability/Arachidonic Acid	0.675	1.72
Thymidine	0.895	2.27
2′-Deoxycytidine	0.865	1.26
Cardiotoxicity Threshold	1.025

#### 2.8.4 Dose-Response Analysis

For the smoke and aerosol bPBS treatments, the bPBS concentration ranges were converted to nicotine concentrations based on the measured nicotine concentration to create a normalized concentration range across samples based on the component analysis. Dose-response analysis was performed using GraphPad Prism (version 9.1, GraphPad Software, San Diego, CA, United States). Each data set was fitted with a nonlinear model. The Akaike information criterion was used to determine if an asymmetric (five-parameter) or multiphasic nonlinear model was a better fit for the data than a four-parameter model. The cardiotoxicity toxicity potential concentration (cTP) was then determined from the prediction model dose-response curve using the cardiotoxicity threshold value (Table 3). The cTP concentration for each sample is provided in both µg/mL nicotine and % bPBS (which could be used to determine the concentration of other constituents).

An extra sum-of-squares F test was used to determine if the dose-response curve of each endpoint (cell viability, lactic acid, thymidine, arachidonic acid, and 2′-deoxycytidine) for the cigarette reference sample (1R6F bPBS) were significantly different from each other, under the null hypothesis that one curve adequately fits all data sets (i.e., the data sets have the same top, bottom, IC_50_, and hill slope best-fit values), and the alternative hypothesis that there is a different dose-response curve for each data set (i.e., the data sets have different top, bottom, IC_50_, and Hillslope best-fit values).

If it was determined that the data sets had independent dose-response curves, an unpaired t test was conducted for each endpoint at each closest or overlapping concentration to determine the concentration where the curves differed from one another. The resulting *p* values were adjusted for multiple comparisons (*q* value) using Benjamini and Hochberg’s method to control the false discovery rate. These analyses were also used to determine if the dose-response curves for the 1R6F bPBS was significantly different from the NGP bPBS and samples tested in the same experiment (e.g., 1R6F S1 was compared to cHTP, EVP-FB, EVP-0, and EVP-NS; 1R6F S2 was compared to pHTP1, pHTP2). Additionally, the response following cHTP bPBS exposures were compared to EVP-0, EVP-FB, and EVP-NS bPBS. The significance threshold was set at *p* < 0.05 for all statistical tests. All values in figures are given as mean ± standard error. If the standard error is not shown, the error bars are smaller than the size of the symbol.

## 3 Results

### 3.1 Characterisation of bPBS for Reference Cigarette and NGPs

Nicotine and eight carbonyls were quantified in the bPBS matrix following the capture of the 1R6F smoke and the individual NGP aerosols. Quantification results are displayed as µg/mL of bPBS in Table 4. The 1R6F S1 and S2 bPBS samples contained the highest level of carbonyls. By contrast, the total quantified carbonyls were greatly reduced or not quantifiable in the NGP samples. Lower levels were detected for the HTP samples compared to 1R6F for all eight quantified carbonyls, whereas the majority of these were below the LOQ for the EVP bPBS samples. Formaldehyde levels in the EVP-NS bPBS samples were higher compared to the seven other carbonyls but did not exceed those measured in both 1R6F bPBS samples. The EVP-NS delivered the highest level of nicotine to the PBS, with both EVP-NS and EVP-FB bPBS containing more nicotine than all three HTPs and 1R6F bPBS samples. Low levels of nicotine (0.5 μg/mL) were detected in the EVP-0 bPBS, which was not recorded in the neat e-liquid sample, as per the Certificate of Analysis (determined using gas chromatography).

**TABLE 4:** Nicotine and carbonyl content (µg/mL) of test article smoke or aerosols used in this study were measured using LC-MS/MS and HPLC-DAD, respectively.

Concentration (µg/mL)	1R6F S1	1R6F S2	cHTP	pHTP1	pHTP2	EVP-FB	EVP-NS	EVP-0	LOQ (µg/mL)
Nicotine	127.1	152.0	123.0	101.0	140.0	165.1	187.0	0.5	0.01
Formaldehyde	12.6	13.1	0.9	0.6	0.8	5.0	9.0	4.4	0.25
Acetaldehyde	190.3	171.4	52.9	53.3	50.5	<LOQ	3.3	<LOQ	1.5
Acetone	55.8	21.76	5.4	5.1	5.5	<LOQ	<LOQ	<LOQ	1.0
Acrolein	4.0	5.0	1.3	<LOQ	0.4	<LOQ	<LOQ	<LOQ	0.5
Propionaldehyde	10.5	9.1	3.5	2.6	2.6	<LOQ	<LOQ	<LOQ	0.5
Crotonaldehyde	8.3	4.9	0.6	0.9	0.8	<LOQ	<LOQ	<LOQ	0.5
2-Butanone	11.9	4.8	1.3	1.1	1.1	<LOQ	<LOQ	<LOQ	0.5
n-Butyraldehyde	4.0	3.28	2.8	2.8	2.7	<LOQ	<LOQ	<LOQ	0.5

### 3.2 Nicotine Cardio^*qP*^ Results

**FIGURE 1:**
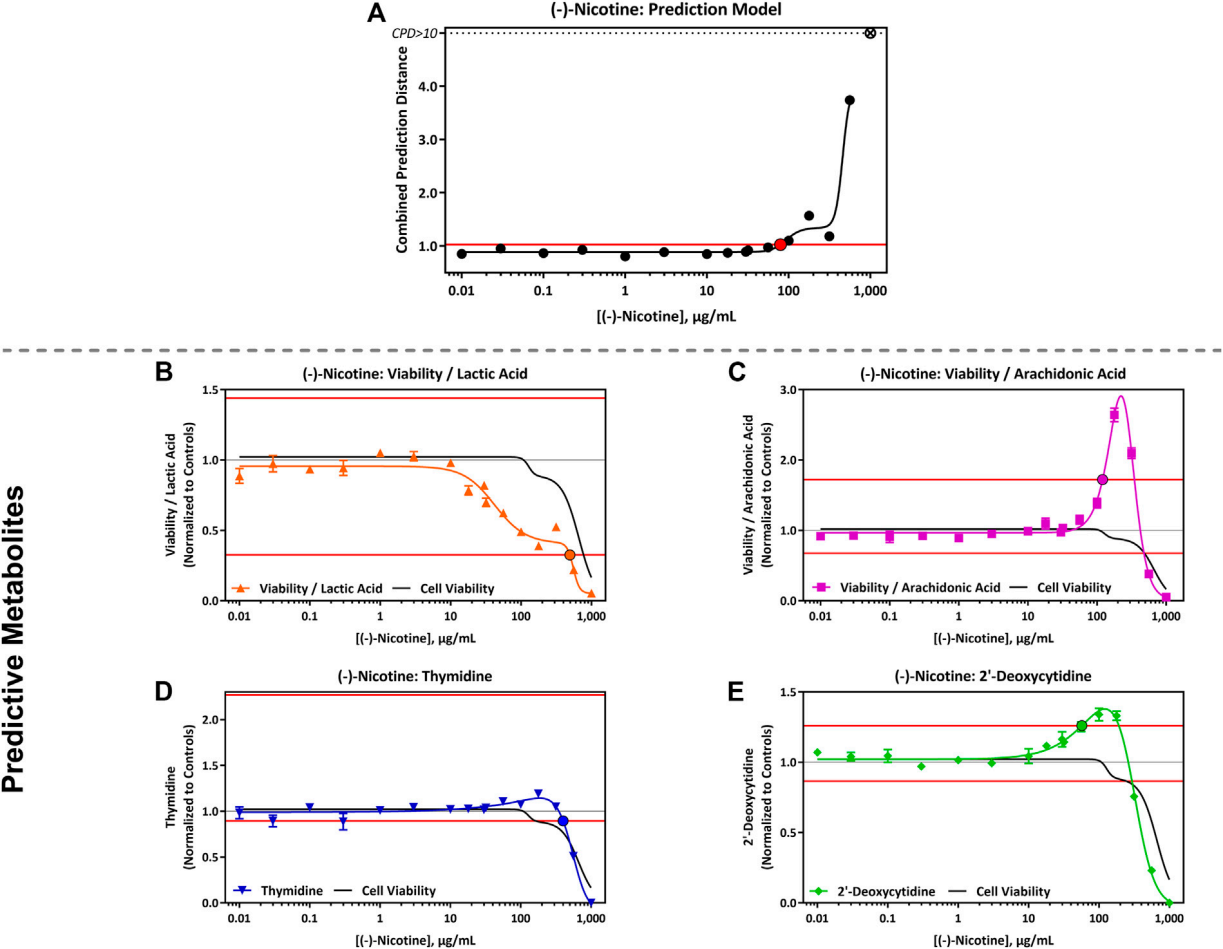
Cardio^qP^ Assay Results Following Exposure to 0.01–1,000 μg/mL (-)-Nicotine. **(A)** (-)- Nicotine dose-response results for the Cardio^qP^ prediction model. The horizontal red line represents the cardiotoxicity threshold and the red filled circle indicates the predicted cardiotoxicity potential concentration (cTP). Concentrations greater than the cTP are predicted to be cardiotoxic. The y-axis is the combined prediction distance, which is determined from each biomarker ratio’s response normalized to its predictive threshold (level of change required for a toxic prediction). CPD: Combined prediction distance; ⊗: CPD>10 **(B)** Change in Cell Viability/Lactic Acid; **(C)** Cell Viability/Arachidonic Acid; **(D)** Thymidine, and **(E)** 2′-Deoxycytidine. The horizontal red lines represents the metabolite-specific prediction thresholds. The y-axis is the reference treatment normalized (fold change) value for each metabolite/ratio. The black bordered circles represent the nicotine concentration where the dose-response curve crossed the metabolite-specific prediction threshold.

The Cardio*qP* prediction model was based on the perturbation of four key metabolites (lactic acid, arachidonic acid, thymidine and 2′-deoxycytidine) and cell viability in hiPSC-CM. The predicted cTP concentration for nicotine was 80 μg/mL and viability IC50 was 586 μg/mL (Table 5). Nicotine caused metabolic perturbations in all four predictive metabolites (Figure 1), with viability/lactic acid responding at the lowest concentrations, but viability/arachidonic acid and 2′-deoxycytidine crossing their respective prediction thresholds first. Viability/lactic acid ratio showed a downward trend after 10 μg/mL of nicotine, and the fold change continued to decrease as the concentration of nicotine increased. At 495 μg/mL, the fold change dropped below the lower metabolite-specific prediction thresholds. Viability/arachidonic acid ratio displayed a different trend to viability/lactic acid. Initially, an upward trend was observed after 50 μg/mL of nicotine, and the fold change continued to increase, exceeding the upper metabolite-specific prediction threshold at 120 μg/mL of nicotine. After 200 μg/mL of nicotine, the fold change decreased as the concentration increased. Thymidine (which dropped below threshold at 398 μg/mL) fold change followed a similar trend as viability/lactic acid (Figures 1B,D), whereas 2′deoxycytidine (which exceeded threshold at 56 μg/ml) was similar to viability/arachidonic acid (Figures 1C,E). Individual cell viability, lactic acid and arachidonic acid dose-response curves can be found in the Supplementary Material (Supplementary Figure S1).

**TABLE 5:** Cardiotoxicity potential summary for smoke or aerosol trapped bPBS from reference cigarettes and NGPs.

Sample bPBS	Concentration range tested (% bPBS)	cTP^a^ (% bPBS)	Viability IC_50_ (% bPBS)	Exposure range (µg/mL nicotine)	cTP^a^ (µg/mL nicotine)	Viability IC_50_ (µg/mL nicotine)
1R6F S1	0.053–3	0.3	1.0	0.07–3.81	0.4	1.3
1R6F S2	0.053–3	0.6	2.3	0.08–4.6	0.9	3.5
cHTP	0.18–10	3.3	10.4	0.22–12.3	4.1	12.8
pHTP1	0.18–10	6.2	ND	0.18–10.1	6.3	ND
pHTP2	0.18–10	7.0	ND	0.25–14.0	9.8	ND
EVP-FB	0.18–10	ND	ND	0.29–16.5	ND	ND
EVP-NS	0.18–10	ND	ND	0.33–18.7	ND	ND
EVP-0	0.18–10	ND	ND	N/A	N/A	N/A
Nicotine	N/A	N/A	N/A	0.1–1000	80	586

a Concentration in % bPBS and μg/mL nicotine where the cardiotoxicity threshold of 1.025 is crossed. cTP: Cardio Toxicity Potential. N/A: Not appropriate. ND: no effect was detected within the exposure range tested.

### 3.3 Cardiotoxicity Potential of bPBS for Reference Cigarette and NGPs

**FIGURE 2:**
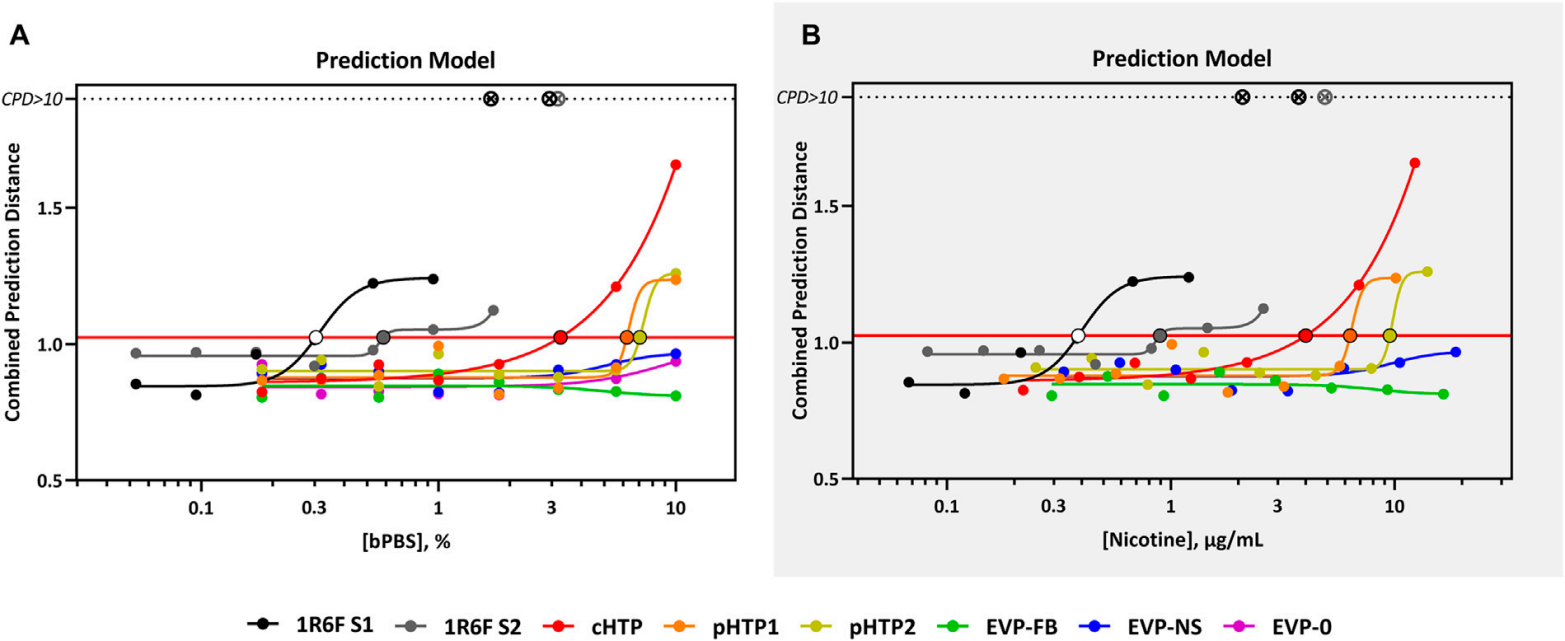
Cardio^qP^ Prediction Model Response Comparison. The horizontal red line represents the cardiotoxicity threshold and the black-bordered, filled circles indicate the predicted cardiotoxicity potential concentration (cTP) for each treatment. The x-axis is the concentration (% bPBS) of the test article **(A)** or nicotine (µg/mL) **(B)**. The y-axis is the combined prediction distance, which is determined from each biomarker ratio’s response normalized to its predictive threshold (level of change required for a toxic prediction). CPD: Combined prediction distance; ⊗: CPD>10.

The predicted cTP concentration and viability IC_50_ for all the test samples are summarized in Table 5 and the dose-response curves for the prediction model are shown in Figure 2. The 1R6F bPBS samples from both studies were predicted to be cardiotoxic: 1R6F S1 at 0.3% (0.4 μg/ml of nicotine) and 1R6F S2 at 0.6% (0.9 μg/ml of nicotine). Based on reproducibility testing (data not shown), the variation between S1 and S2 were within the expected level (i.e., within 3-fold, a value determined by Stemina as not biologically different). All three HTP bPSB samples elicited a metabolic response indicative of cardiotoxicity in this assay. However, the predicted cTP concentrations for the HTPs samples were approximately 10-fold higher than the corresponding 1R6F sample, indicating that 1R6F bPBS has the potential to cause cardiotoxicity at a concentration that was 10-fold more potent than the HTP bPBS samples. By contrast, the three EVP bPBS (EVP-0, EVP-FB, and EVP-NS) did not cause a metabolic response indicative of cardiotoxicity potential within the exposure concentrations tested. The cTP (µg/mL nicotine) were then compared to the maximum blood nicotine concentration (Cmax) observed in adult smokers following cigarette smoking (16 ± 9 ng/mL) (D'Ruiz et al., 2015) reported in Table 6. An *in vitro* response observed at ≤ 50 × the *in vivo* Cmax was considered to be relevant for prediction of *in vivo* toxicity (Talbert et al., 2015). The prediction model dose-response curves for each test article were statistically compared with one another for the treatments that were conducted within the same study (extra sum-of-squares F Test). When comparing the bPBS curves, both 1R6F bPBS (S1,2) were significantly different from all the NGP bPBS samples (*p* < 0.0001) see Table 7 and individual values for each product and the four metabolites in Supplementary Tables S1 to S14; the only two pairs which were not significantly different to each other were: pHTP1 and pHTP2, and EVP-NS and EVP-0*.*

**TABLE 6:** Cardiotoxicity potential fold change relative to typical nicotine concentrations following smoking a cigarette.

Sample bPBS	cTP (µg/mL nicotine)	cTP vs C_max_ (0.016 μg/mL ^a^)
1R6F S1	0.4	25
1R6F S2	0.9	56
cHTP	4.1	256.35
pHTP1	6.3	394
pHTP2	9.8	612.5
EVP-FB	ND	N/D
EVP-NS	ND	N/D
EVP-0	N/A	N/A
Nicotine	80	5000

a D'Ruiz et al.(2015).

N/A: not appropriate. ND: no effect was detected within the exposure range tested.

*In vitro* response observed at ≤ 50 × the *in vivo* C_max_ is considered to be relevant for prediction of *in vivo* toxicity (Talbert et al., 2015).

**TABLE 7:** Prediction model of the F test comparison (*p* values) for the product dose response curves, relative to %bPBS or nicotine (μg/mL).

bPBS (%)								Nicotine (µg/ml)					
	1R6F S1	1R6F S2	cHTP	pHTP1	pHTP2	EVP-FB	EVP-NS	1R6F S1	1R6F S2	cHTP	pHTP1	pHTP2	EVP-FB
1R6F S2	<0.0001							<0.0001					
cHTP	<0.0001							<0.0001					
pHTP1		<0.0001							<0.0001				
pHTP2		<0.0001		0.5108					<0.0001		0.4806		
EVP-FB	<0.0001		<0.0001					<0.0001		<0.0001			
EVP-NS	<0.0001		<0.0001			<0.0001		<0.0001		<0.0001			0.0002
EVP-0	<0.0001		<0.0001			0.0011	0.0634						

Treatments that did not have significantly different dose-response curves are highlighted in yellow (p < 0.05). Statistical comparisons were only made on sample run during the same experiment.

#### 3.3.1 Predictive Metabolites

**FIGURE 3:**
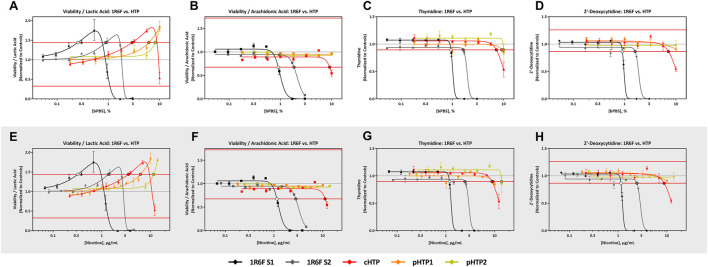
1R6F vs HTP Cell Viability/Lactic Acid, Cell Viability/Arachidonic Acid Thymidine, and 2′-Deoxycytidine Response Comparison. The x-axis is the concentration (% bPBS) of the test article **(A–D)** or nicotine (µg/mL) **(E–H)**. The y-axes are the reference treatment normalized (fold change) values for each metabolite/ratio. The horizontal red lines represent the metabolite-specific prediction thresholds The black bordered circles represent the nicotine concentration where the dose-response curve crossed the metabolite-specific prediction threshold.

**FIGURE 4:**
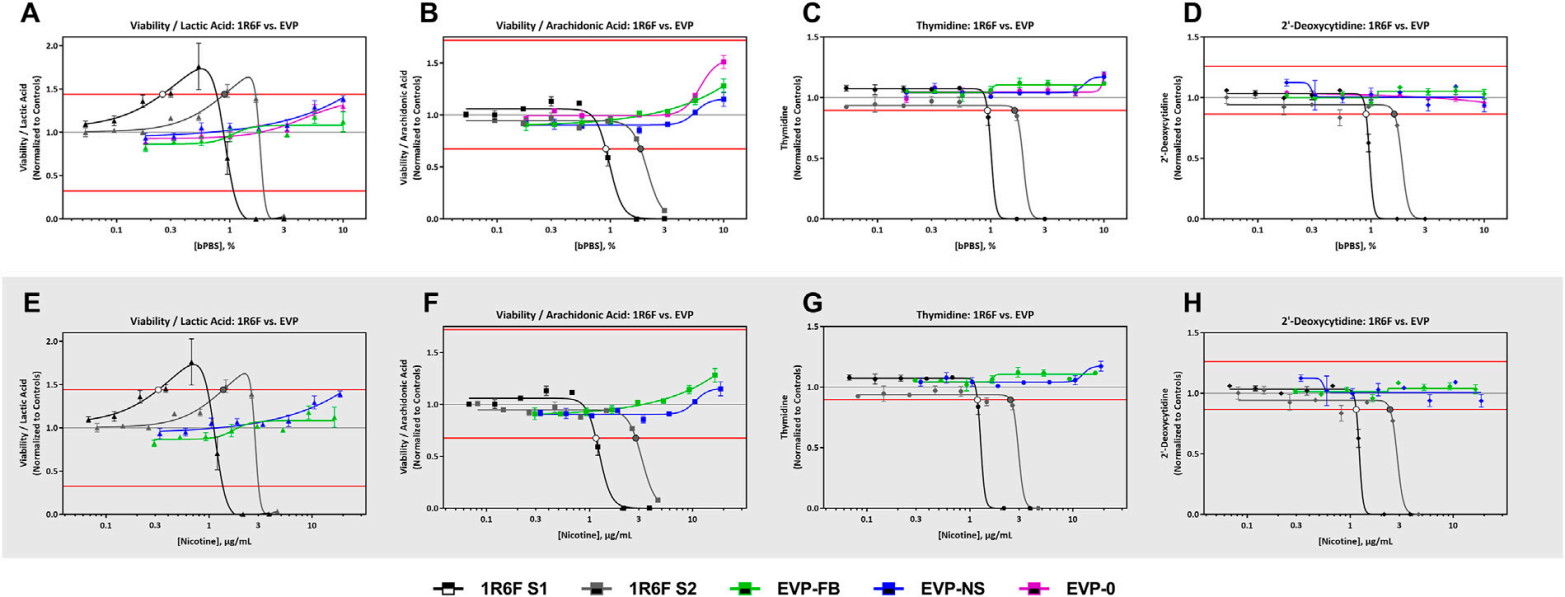
1R6F vs EVPs Cell Viability/Lactic Acid, Cell Viability/Arachidonic Acid Thymidine, and 2′-Deoxycytidine Response Comparison. The x-axis is the concentration (% bPBS) of the test article **(A–D)** or nicotine (µg/mL) **(E–H)**. The y-axes are the reference treatment normalized (fold change) values for each metabolite/ratio. The horizontal red lines represent the metabolite-specific prediction thresholds. The black bordered circles represent the nicotine concentration where the dose-response curve crossed the metabolite-specific prediction threshold.

All four predictive metabolites were altered by the 1R6F S1 and S2 bPBS samples Figures 3, 4. Viability/lactic acid was the most sensitive and initially showed a positive dose-response, followed by a decrease, corresponding to decreased cell viability. Viability/arachidonic acid, thymidine and 2′ deoxycytidine decreased as the concentration of bPBS increased. cHTP bPBS caused the same metabolomic response profile as 1R6F bPBS, whereas pHTP 1 and 2 only caused two predictive metabolites (viability/lactic acid and thymidine) to be altered under these study conditions (Figure 3). By contrast, EVP-NS, EVP-FB and EVP-0 caused a slight increase in viability/lactic acid and viability/arachidonic acid; however, this increase did not reach the metabolite-specific cardiotoxicity thresholds at the concentrations tested (Figure 4). Moreover, the three EVP samples did not alter thymidine and 2′deoxycytidine metabolism (Figure 4). Individual viability, lactic acid and arachidonic acid curves can be found in the Supplementary Material (Supplementary Figures S2, S3).

The individual dose-response curves within each predictive metabolites were compared on the basis that all parameters were shared (see *Dose-Response Analysis*) and a point of difference (POD) was determined where applicable (detailed results provided in Supplementary Tables S1–S14. The dose-response curve for each predictive metabolite were significantly different between 1R6F S1 and S2 bPBS (*p* < 0.0001). which was largely due to the shift in concentration where changes was observed between replicates; however, the same response profile was observed. The dose-response curves for the NGP bPBS samples were all significantly different from the corresponding study 1R6F bPBS sample for each endpoint (*p* < 0.0001, determined by an extra sum-of-squares *F* Test, see Supplementary Tables S1–S14). The raw data for nicotine, 1R6f and the NGPS for cell viability and the 4 metabolites are presented in Supplementary Tables S15–S24.

## 4 Discussion

### 4.1 Quantification of Trapped Carbonyls and Nicotine in Bubbled PBS

The eight carbonyls quantified in this study were selected based on a list described by Buratto et al. (2018), which represent the main carbonyl compounds included in the HPHC lists for tobacco smoke regulatory reporting (FDA, 2012; Health Canada, 2000). Nicotine was used in the present study as a comparative measure of exposure between the test samples. There were clear differences between the quantified constituents form the different NGP aerosol and 1R6F bPBS (Table 4). As expected, total carbonyl levels were the highest in the 1R6F bPBS, with substantially lower amounts measured in all HTP bPBS. Limited carbonyls were detected in all EVP bPBS test samples. This is reflective of the whole smoke and aerosol emission profiles typically reported for cigarettes and non-combustible nicotine products (Schaller et al., 2016b; Poynton et al., 2017; Farsalinos et al., 2018; Jaccard et al., 2019; Rudd et al., 2020). For EVP-NS bPBS the formaldehyde level was elevated in comparison to EVP-FB and EVP-0 bPBS, although this was substantially lower than combustible cigarettes based on 120 puffs vs 54 puffs (EVP vs 1R6F).

### 4.2 The Use of hiPSC in Cardiotoxicity Testing

One of the criticisms of using hiPSC-CMs is the fact that they are in embryonic state of development and additional work needs to be done to fully understand the predictive value of this system in the context of preclinical cardiac safety risk assessment. Issues relating to metabolic, structural, and functional maturation are incompletely understood and any of these factors could result in the inconsistent prediction of toxicity across diverse classes of drug candidates when used to screen for new drugs (Pang et al., 2019). However, despite these limitations, due to the increasing prevalence of cardiovascular diseases and the increasing complexity of human exposures to chemicals, it is even more important than ever to consider and adequately test for the potential adverse cardiovascular effects of pharmaceuticals and environmental compounds. However, for drugs and other chemicals, the currently used animal models for cardiovascular endpoints do not provide sufficient information to meet both the high throughput required and human relevance needed, to be able to lower the preventable risks of cardiovascular disease from chemicals (Burnett et al., 2021). The cells used in this assay by Stemina are more metabolically aligned with adult cardiomyocytes, using oxidative phosphorylation instead of glycolysis, which is particularly important as the assay’s cardiotoxicity model is based on changes in the metabolism of key metabolites important for cardiomyocyte function, including lactic acid. Previous research has shown that hiPSC-CM cultured in media containing glucose have a mid-fetal state of energy metabolism, generating ATP primarily through glycolysis (Rana et al., 2012; Correia et al., 2017) however, when the media contains galactose, hiPSC-CM energy metabolism shifts to an oxidative phosphorylation energy source more similar to adult cardiomyocytes (Rana et al., 2012; Correia et al., 2017).

### 4.3 Predictive Metabolites

The four metabolites measured in the Cardio*qP* assay have key roles in modulating oxidative stress, mitochondrial function, and mitochondrial replication, which have been experimentally and clinically associated with cardiotoxicity (described in Palmer et al., 2020) (Adlam et al., 2005; Palmer et al., 2020).

• Lactic acid is known to be associated with cardiotoxicity, especially during cardiac ischemia and heart failure (Sachinidis, 2020). Increased levels of lactic acid in the spent media of hiPSC-CMs signals a change from aerobic respiration to anaerobic respiration and glycolysis.
• 2′-deoxycytidine and thymidine are two of the principal DNA nucleosides and key components of the pyrimidine metabolism pathway. Changes in the metabolism of these metabolites is indicative of mitochondrial toxicity, which is an underlying mechanism of cardiotoxicity (reviewed in Varga et al., 2015)
• Arachidonic Acid is a polyunsaturated, essential fatty acid that is involved in lipid transport, metabolism, and fatty acid metabolism. Arachidonic acid release and oxygenation by enzyme systems leads to the formation of eicosanoids, which is an important group of inflammatory mediators and contributes to the pathogenesis of cardiovascular disease (Sonnweber et al., 2018).

Palmer et al. (2020) tested a panel of known functional and structural cardiotoxicants. In that study, functional cardiotoxicants generally “*caused an increase in lactic acid independent of* (*or prior to*) *changes in the other biomarkers*,” whereas “*changes in arachidonic acid, 2′ deoxycytidine, and thymidine prior to changes in lactic acid occurred following exposure to many of the structural cardiotoxicants tested*.” Lactic acid and arachidonic acid are present in the cell culture medium, the change detected indicates that they were secreted (response >1) or taken-up (response <1) by hiPSC-CMs. Thymidine and 2′deoxycytoinde were not media components, therefore elevated levels in response to test article treatment indicates an increase in the amount secreted by hiPSC-CMs, whereas a decrease indicates that lower levels were secreted.

### 4.4 Nicotine

Like other mild stimulants, it has been reported that nicotine can cause transient cardiovascular effects in humans at physiological levels. Nicotine acts by binding to nicotine acetylcholine receptors, activating the sympathetic nervous system which leads to vasoconstriction and increases in heart rate, blood pressure, and myocardial contraction (Benowitz and Burbank, 2016). In this study, we assessed whether neat nicotine alters any of the key metabolites assessed in this assay. Low concentrations of nicotine (0.01–10 μg/mL) did not alter the four metabolites; however, all four metabolites were impacted at higher concentrations. Lactic acid was the most sensitive metabolite measured causing a dose-dependent increase at ≥10 μg/ml (Supplementary Figure S1). This change was prior to the other biomarkers, therefore, as mentioned previously (*Predictive Metabolites*), at high concentrations (supraphysiological), and under the conditions of test, neat nicotine may have functional cardiotoxic potential. Winbo et al. (2020) demonstrated that when hiPSC-CM monocultures were exposed to nicotine (1 µM = 0.162 μg/mL), the functional endpoint, beating rate, significantly decreased indicating that nicotine can modulate the contractility function of hiPSC-CM (Winbo et al., 2020).

Nicotine exposure induced a small increase in thymidine and 2′-deoxycytidine, indicating that nicotine may cause mitochondrial toxicity at higher concentrations. A recent study by Jia et al. (2020) found that chronic nicotine exposure impaired mitochondrial function, resulting in intrinsic apoptosis, which is consistent with the changes observed in this study prior to decreased cell viability.

Neat nicotine was predicted to be cardiotoxic at 80 μg/mL (493 μM, see Table 6) in this specific assay, however the cTP was approximately 5000 times higher than the C_max_ observed after smoking a cigarette (16 ± 9 ng/mL) in adult smokers (D'Ruiz et al., 2015). Based on the results of this study, nicotine is not predicted to be cardiotoxic at physiological levels achieved after smoking a cigarette or use of the assessed NGPs. Moreover, the cTP was not considered relevant *in vivo* as the predicted dose was supraphysiological, and these blood concentrations could not be reached during normal use of the products. The high cTP predicted (80 μg/mL) also suggest that nicotine was not the predominant driver of cardiotoxicity observed for 1R6F (S1 and 2) and HTP (cHTP, pHTP 1 and 2) bPBS as the nicotine concentration tested did not exceed 14 μg/mL.

### 4.5 Potential Cardiotoxicity Screening for Reference Cigarettes When Compared to NGPs

As reported above, 1R6F altered hiPSC-CM metabolism at lower concentrations and exhibited a different metabolic response compared to either the HTP or EVP variants (Figures 3, 4). The 1R6F bPBS samples caused a significant decrease in all metabolites, with changes to viability/lactic acid being observed prior to changes in other metabolites. Lactic acid is known to be associated with cardiotoxicity, especially during cardiac ischemia and heart failure (Sachinidis, 2020). The 1R6F bPBS potentially acted as a functional cardiotoxicant, based on the predictions of Palmer et al. (2020); however, it is important to note that most of the drugs known to cause functional cardiotoxicity initially caused an increase in lactic acid prior to changes in cell viability, in contrast to the decrease observed here. A similar trend was also seen for the HTP samples but importantly at much higher bPBS concentrations (10-fold). Additionally, these changes in viability/lactic acid were not the same pattern observed with neat nicotine. For nicotine there was no initial uptake of lactic acid at lower concentrations. The cTP of nicotine was 80 μg/mL indicating that nicotine was not the driver of the cardiotoxic potential observed in this assay, and likely it is other smoke constituents generated by tobacco combustion that drive cardiotoxicity. The 1R6F and cHTP bPBS samples both caused a decrease in viability/arachidonic acid at higher concentrations, whereas both the pHTP bPBS samples did not impact this ratio at the concentrations evaluated in this study. The 1R6F bPBS decreased thymidine at concentrations 5–10 times lower than the HTP samples. Lastly, 1R6F and cHTP decreased 2′-deoxycytidine, with cHTP being 4-8-fold less potent than 1R6F. The alterations of these two amino acids thymidine and 2′-deoxycytidine at lower concentrations for 1R6F vs HTP could reflected the higher oxidative stress reported for 1R6F vs HTP and associated mitochondrial toxicity (Malinski et al., 2018).

The three HTP bPBS samples were tested in two separate studies: cHTP in study 1 and pHTP 1 and 2 in study 2. Overall, the cHTP bPBS appeared more potent than both pHTP samples for all endpoints and had a slightly lower cTP concentration; however, the concentrations predicted to be cardiotoxic were within 3-fold of each other and therefore not considered to be biologically different based on expected variability of the assay (Palmer et al., 2020, personal communication, 14th June 2021). Importantly, long term *in vivo* and clinical studies with adult smokers that have compared commercialised HTPs with combustible cigarette smoke exposure, have shown significant improvements in cardiovascular endpoints and related biomarkers upon switching from combustible cigarettes to HTPs (Sakaguchi et al., 2014; Ogden et al., 2015; Smith et al., 2016; Schaller et al., 2016b; Schaller et al., 2016a; Pratte et al., 2017; Gale et al., 2019; Phillips et al., 2019; Shein and Jeschke, 2019; Tran et al., 2020; Wong et al., 2020). A recent systematic review of the health effects of HTP by Zynk et al. (2021) reported favourable improvements in biomarkers related to cardiovascular and respiratory endpoints when comparing continued combustible cigarette smoking adult smokers who switched to using HTPs (Znyk et al., 2021). Improvements in clinically relevant risk markers, especially cholesterol, sICAM-1, 8-epi-PGF2α, 11-DTX-B2, HDL and FEV1 have been observed when adult smokers switched away from combustible cigarettes to HTPs (Zynk et al., 2021).

All three EVP bPBS test samples (EVP-0, EVP-FB, and EVP-NS) did not cause a metabolic response indicative of cardiotoxicity potential at the highest exposure level tested in the current study (10% bPBS) (Figure 2 and Table 5). This may not mean that these products are benign in all biological systems, but the levels of chemicals within the complex mixtures delivered to the cells in this study were not high enough to elicit a response. However, the EVP samples have been demonstrated as potentially reduced risk for this *in vitro* endpoint when compared to the responses seen from HTP and 1R6F samples. An increase in cell viability/arachidonic acid ratio was observed which was the opposite to that seen for 1R6F and HTP bPBS samples (Supplementary Figures S2, S3), suggesting that 1R6F and HTP bPBS may alter the metabolism of hiPSC-CMs differently to that of EVP bPBS. A cross-comparison of the prediction model dose-response curves between the three EVP bPBS identified a significant difference between EVP-FB and the other two EVP samples; however, EVP-NS and EVP-0 were not significantly different.

Malinska et al. (2018) compared 3R4F and THS2.2 (IQOS) Total particulate matter (TPM) exposed to BEAS2B cells for up to 12 weeks, looking for markers of oxidative stress. The authors reported that the amount of TPM that needed to cause a similar extent of oxidative stress and changes in cellular mitochondrial energy production, was 20-fold higher for THS 2.2 (150 μg/mL vs 7.5 μg/ml 3R4F). TPM from THS2.2 at this higher concentration exerted similar effects on protein carbonylation; mitochondrial superoxide levels, and levels of GPx1. Gene expression analysis was also reported to show similar changes in expression patterns of key oxidative stress response genes in BEAS2B cells exposed to either 7.5 μg/mL TPM from reference 3R4F or 150 μg/mL of TPM from THS2.2 aerosol. In the current study, for the three HTP products there was approximately a 10-fold decrease in response when compared to the response to 1R6F. This may also reflect the potential reduction of oxidative stress seen with these products. In this study, The comparison of aqueous extracts was made using 56 puffs of 1R6F vs 120 puffs for the NGPs. This equates to a difference of 20-fold when the different puff numbers are taken in to account. Munakata et al. (2018) also reported reductions in the GSH/GSSG ratio observed in BEAS2B cells exposed to aqueous extracts (AqE) of an HTP product, NTV, and EVP exposures at higher effective concentrations than with observed reductions following 3R4F exposure. This reduction in GSH/GSSG followed the same trend of relative product effects as seen in the current study: 3R4F > HTP > EVP in terms of antioxidant activity.

Oxidative stress and activation of the NRF2/antixodiant response element (ARE) pathways are known to be related to inflammatory responses (Gilmour et al., 2003) and have been seen in human bronchial epithelial cells exposed to cigarette smoke (Taylor et al., 2016). Cigarette smoke AqE exposure has been reported to cause a dose-dependent decrease in the ratio of reduced glutathione to oxidized glutathione (rGSH/GSSG) together with an increased translocation of Nrf2 to the nucleus demonstrated by Western blot analysis. Knock down of the Nrf2 pathway by siRNA completely blocked cigarette smoke AqE -induced IL-8 cytokine release (Lau et al., 2012). In a paper by Czekala et al. (2021), it was demonstrated that repeated exposure of NHBE cells to undiluted EVP aerosol at 30, 60 or 90 puffs for 28 days puffs saw no elevation of IL-8 levels, compared to the strong IL-8 response observed to 90 puffs of 1:17 diluted 3R6F smoke. These relative effects of the two different product categories are in line with the findings of the current study.

Reduced cardiovascular effects have also been reported in longer term *in vivo* and clinical studies with people switching from cigarettes to EVPs, including substantial reductions in biomarkers of exposure to toxicants (including cardiovascular toxicants) in adult smokers who switch from combustible cigarettes to EVP (O'Connell et al., 2016; National Academies of Sciences et al., 2018; Hasan et al., 2020; Abouassali et al., 2021). Moreover, a recent British Heart Foundation funded clinical study found that adult smokers who switched to EVP for 1 month showed favourable improvements in cardiovascular endpoints with significant improvements in endothelial function, arterial stiffness and systolic blood pressure compared with continuing smoking (George et al., 2019). Zynk et al. (2021) stated that HTP has reduced cytotoxicity when compared to smoking but no effect on cytotoxicity was seen with EVPs, which is what was seen with the cell viability/lactic acid graphs. Zynk et al. (2021) reported that cardiovascular risk associated with EVP use is lower than risk associated with combustible cigarette smoking, but may be a higher risk for people with a predisposal cardiovascular disease (Znyk et al., 2021). The results from the present study would support this.

### 4.6 Use of Cardio*qP* Assay as Part of a Weight of Evidence Approach

This assay was implemented as part of a wider framework of pre-clinical *in vitro* toxicity testing (see Czekala et al. (2021) for a brief outline), which is aligned with the principles of the 3Rs and the 21st Century Toxicology framework, which aim to minimise, or remove where possible, experimental animal use (Berg et al., 2011; Rovida et al., 2015). The framework aims to increase human relevance where possible by using human-derived cell (primary or immortalised) -based *in vitro* assays. These assays include, for example, the DiscoverX BioMap platform to screen for human smoking-related disease relevant biomarkers (Simms et al., 2021). This assay uses an extensive panel of human primary cells, including single cell and co culture systems, and changes in molecular pathways linked to cardiovascular toxicity can be inspected. In the Simms et al. (2021) study, reductions in (cardio) toxicity readouts were observed for NGPs when compared to combustible cigarettes. Another cardiovascular toxicity-related assay within the framework is the scratch wound assay, which looks at the rate of repair of human umbilical endothelial cell layers after a “wound” is cut, and can indicate wound healing rates following exposure to NGP samples relative to combustible cigarette smoke extracts (Simms and Trelles, 2019; Chapman et al., 2021). Results need to be confirmed with human clinical studies, which include assessment of short term safety following use of the test products, including cardiovascular parameters (O’Connell et al., 2019; Chapman et al., 2021; Morris, 2021 under review). Looking at the data from such pre-clinical and clinical assessments in combination can provide weight of evidence, which can be further compared to the scientific literature, to draw conclusions on the potential effects of NGPs relative to combustible cigarette smoking. Observation of the same trends across multiple studies gives scientists more confidence in the reduced risk potential of NGPs when compared to combusted products. It is believed that the Cardio*qP* assay adds significantly to the battery of information that we collect on potential cardiotoxic effects of NGPs, and in comparison to combustible cigarette.

### 4.6 Study Limitations

The results of the present study should be viewed within the context of its limitations. The prediction model is currently not able to differentiate functional from structural cardiotoxicity potential with complex mixtures, which may be partially attributed to mixtures having both functional and structural cardiotoxic elements to their toxicity profiles. To date this model has had limited use with complex mixtures. To ensure reproducibility, and therefore valid outcomes in the assay, standardised (ISO) puffing regimes and fixed puff numbers were implemented across studies 1 and 2. All studies were not conducted at the same time, however, the relative difference between the 1R6f and HTP, cTP values were consistent across the studies. This indicates reproducibility of the data outputs for this assay. Furthermore, for trapping of smoke/aerosols, it is difficult to find an ideal medium which could both trap smoke constituents as well as aerosol particulates and gas phase, and be able to be added to the cell culture. For that reason, bPBS was chosen as this could be used in a range of cell culture assays; however its limitations are acknowledged, including the reduced trapping potential of hydrophobic compounds (Smart and Phillips, 2021). It is also important to note that no single *in vitro* assay can fully model the biological complexity of cardiovascular function and disease. However, the Cardio^*qP*^ assay is a useful screen for short term cardiotoxicity and should complement other pre-clinical and clinical techniques for determining the potential cardiovascular impact of NGPs relative to that seen for combustible cigarettes.

## 5 Conclusion

The aim of the present study was to predict the cardiotoxicity potential of NGPs when compared to cigarettes in a human relevant cardiac cells and to demonstrate the utility of this assay as a potential screening tool for this endpoint. Burnett et al. (2021) stated that the use of hiPSC-CMs fills a critical gap where no routine testing for cardiotoxicity is currently performed (Burnett et al., 2021). The Cardio*qP* assay, which is human relevant, low cost, and high-throughput, was able to detect potential cardiotoxic effects of both the combustible cigarettes samples and all the HTP products when hiPSC-CMs were exposed to bPBS trapped samples for 72 h. However, the cTP responses to the 1R6F bPBS samples were significantly greater, with combustible cigarettes eliciting this response at concentrations 10-fold lower than for HTP. HTP bPBS samples also had a different metabolite response profile when compared to 1R6F bPBS, indicating these non-combustible tobacco-containing products are a different product category to combustible cigarettes. All the EVP bPBS samples were negative for cardiotoxic potential in the assay when tested at concentrations up to 10% bPBS under the assay conditions, this does not mean that they are benign but that the concentrations tested were not reactive enough in this assay. The testing of neat nicotine in isolation suggests that due to the much higher concentrations required, the observed effects following exposure to 1R6F smoke and HTP aerosol samples were not driven by nicotine but more likely by other constituents present in cigarette smoke or HTP aerosol. The nicotine cTP was approximately 100–200 times higher than the cTP 1R6F and 9–13 times higher than cTP for HTPs. In summary, the Cardio*qP* assay was able to differentiate between the these three product categories. Overall, the outcomes of the present study add to this growing body of scientific evidence indicating that NGPs have the potential to be less harmful to the cardiovascular system than continued combustible cigarette smoking and in terms of cardiotoxic potential in this assay Clearly no one *in vitro* assay can model all aspects of cardiovascular disease. However, this assay adds to the information we hold on the *in vitro* toxicity of an acute exposure to bPBS of 1R6F, HTP, and EVP.

## Data Availability

The original contributions presented in the study are included in the article/Supplementary Material, further inquiries can be directed to the corresponding authors.
