# Supplementary material for: Use of Human Induced Pluripotent Stem Cell-Derived Cardiomyocytes to Predict the Cardiotoxicity Potential of Next Generation Nicotine Products
Source: Front Toxicol. 2022 Feb 16;4:747508. doi: 10.3389/ftox.2022.747508 (PMC8915889; doi:10.3389/ftox.2022.747508)
Supplement: Supplementary file 1 [file DataSheet1.docx]

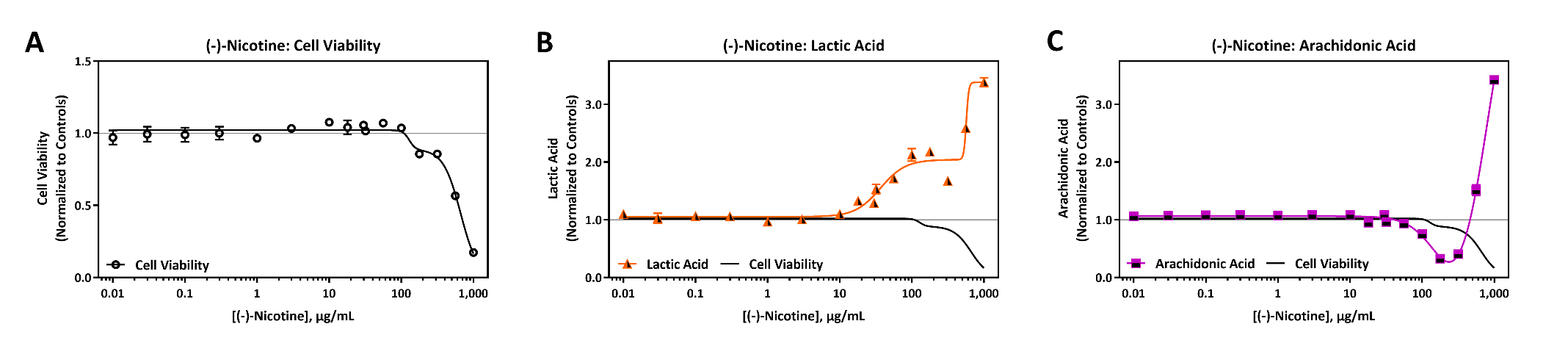


Supplementary Figure S1: Cell viability, Lactic Acid and Arachidonic Acid response curve of (-)- Nicotine

*The x-axis is the concentration of nicotine (µg/mL)* ***(A-C))****.The y-axes are the reference treatment normalized (fold change) values for each endpoint.*


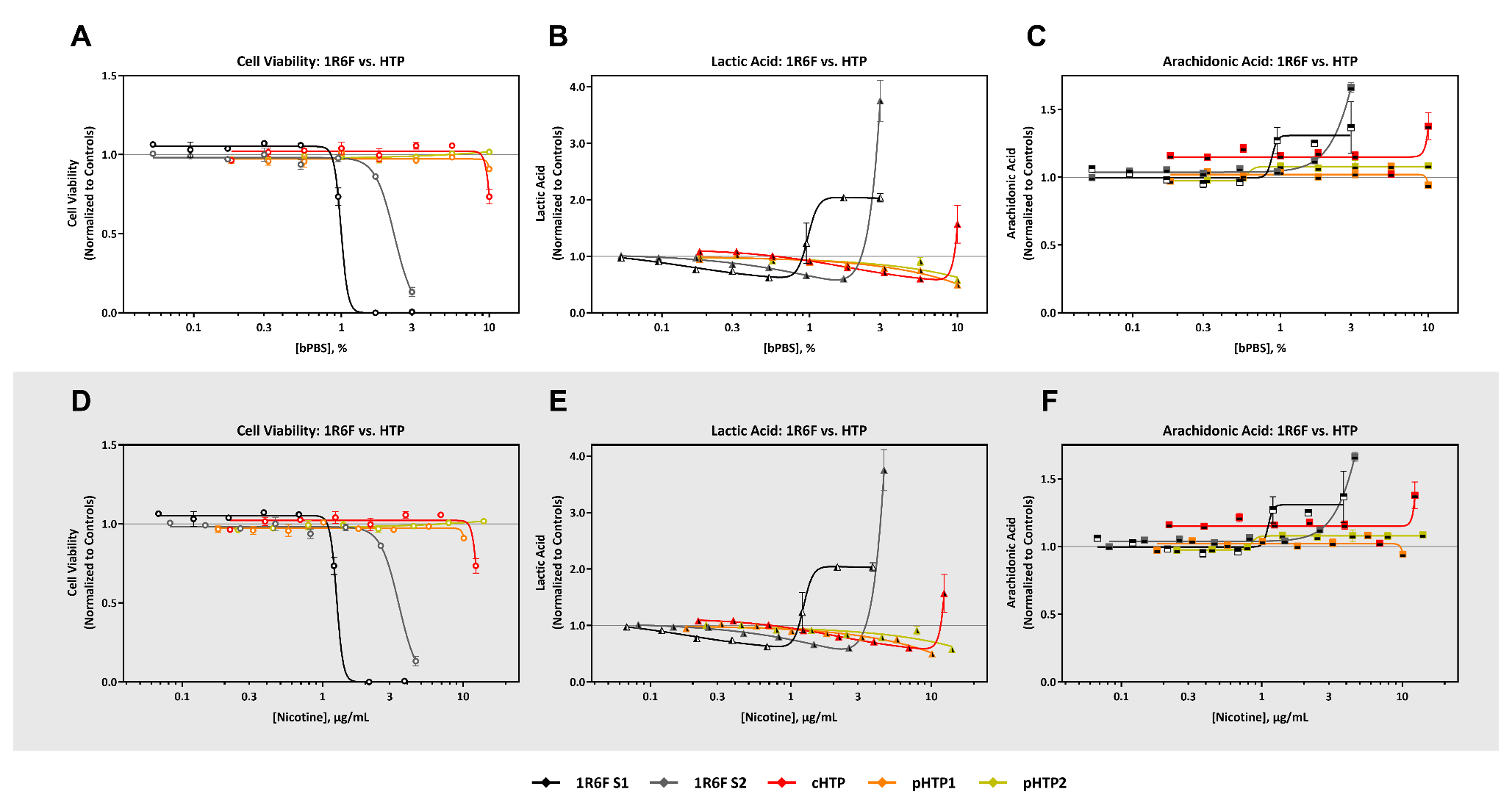


Supplementary Figure S2: 1R6F vs HTP Cell Viability, Lactic Acid, and Arachidonic Acid Response Comparison

*The x-axis is the concentration (% bPBS) of the test article* ***(A-C)*** *or nicotine (µg/mL)* ***(D-F))****.The y-axes are the reference treatment normalized (fold change) values for each endpoint.*


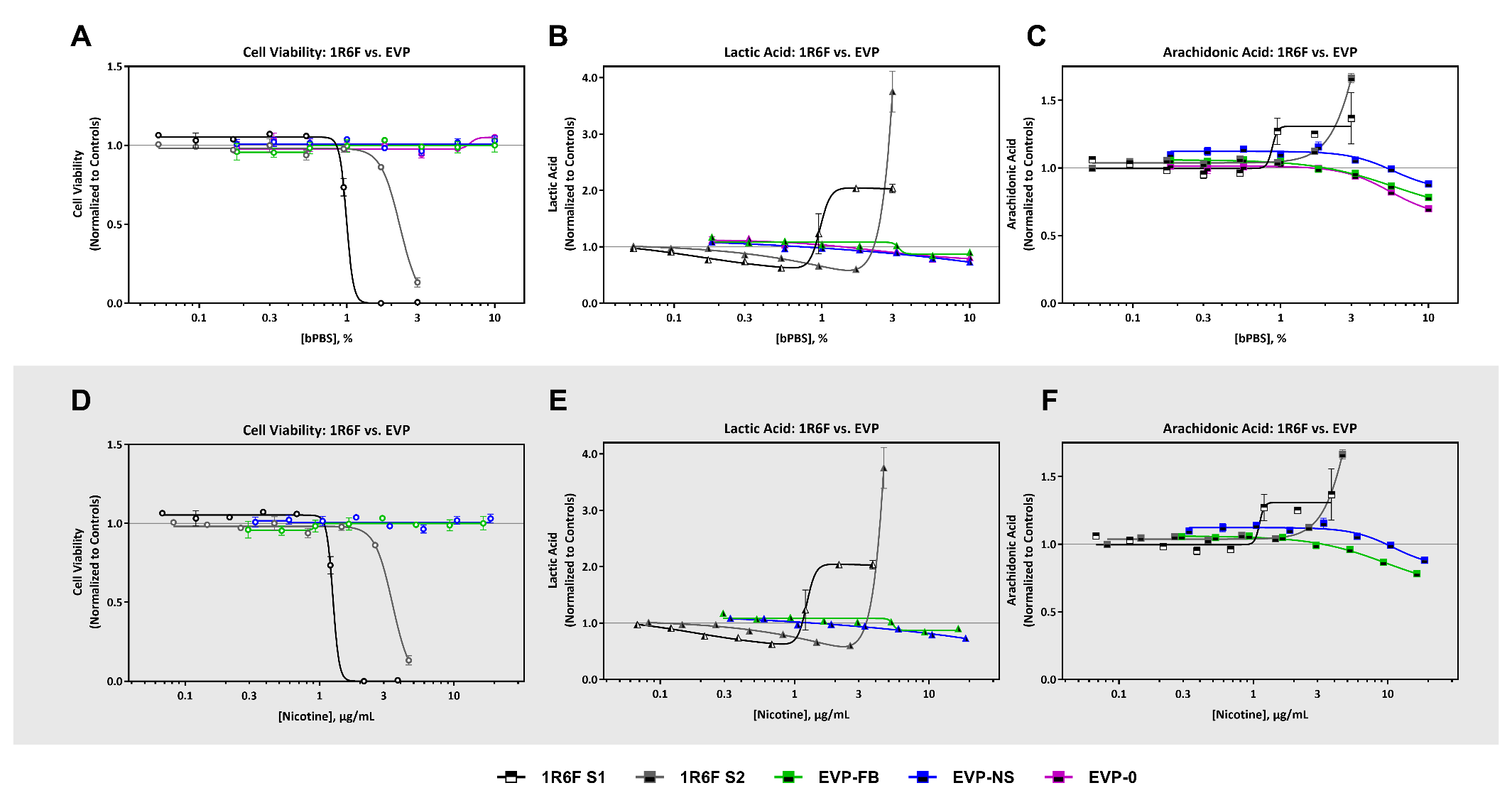


Supplementary Figure S3: 1R6F vs EVP Cell Viability, Lactic Acid, and Arachidonic Acid Response Comparison

*The x-axis is the concentration (% bPBS) of the test article* ***(A-C)*** *or nicotine (µg/mL)* ***(D-F))****.The y-axes are the reference treatment normalized (fold change) values for each endpoint.*

Supplemental Table S1: Viability/Lactic Acid Dose-Response Curve F Test Results (p-values)

| ***bPBS (%)*** | | | | | | | | ***Nicotine (µg/mL)*** | | | | | | |
| --- | --- | --- | --- | --- | --- | --- | --- | --- | --- | --- | --- | --- | --- | --- |
|  | ***1R6F S1*** | ***1R6F S2*** | ***cHTP*** | ***pHTP1*** | ***pHTP2*** | ***EVP-FB*** | ***EVP-NS*** | ***1R6F S1*** | ***1R6F S2*** | ***cHTP*** | ***pHTP1*** | ***pHTP2*** | ***EVP-FB*** |  |
| ***1R6F S2*** | <0.0001 |  |  |  |  |  |  | <0.0001 |  |  |  |  |  |  |
| ***cHTP*** | <0.0001 | <0.0001 |  |  |  |  |  | <0.0001 | <0.0001 |  |  |  |  |  |
| ***pHTP1*** | <0.0001 | <0.0001 | <0.0001 |  |  |  |  | <0.0001 | <0.0001 | <0.0001 |  |  |  |  |
| ***pHTP2*** | <0.0001 | <0.0001 | <0.0001 | 0.5517 |  |  |  | <0.0001 | <0.0001 | <0.0001 | 0.0024 |  |  |  |
| ***EVP-FB*** | <0.0001 | <0.0001 | <0.0001 | <0.0001 | <0.0001 |  |  | <0.0001 | <0.0001 | <0.0001 | <0.0001 | <0.0001 |  |  |
| ***EVP-NS*** | <0.0001 | <0.0001 | <0.0001 | 0.001 | 0.0365 | 0.0038 |  | <0.0001 | <0.0001 | <0.0001 | <0.0001 | 0.0014 | 0.0099 |  |
| ***EVP-0*** | <0.0001 | <0.0001 | <0.0001 | <0.0001 | 0.0009 | 0.1179 | 0.3473 |  |  |  |  |  |  |  |

Treatments that did not have significantly different dose-response curves are highlighted in yellow (p<0.05).

Supplemental Table S2: Points of departure (POD): Viability/ Lactic acid

| ***POD bPBS (%)*** | | | | | | | | ***POD Nicotine (µg/mL)*** | | | | | | |
| --- | --- | --- | --- | --- | --- | --- | --- | --- | --- | --- | --- | --- | --- | --- |
|  | ***1R6F S1*** | ***1R6F S2*** | ***cHTP*** | ***pHTP1*** | ***pHTP2*** | ***EVP-FB*** | ***EVP-NS*** | ***1R6F S1*** | ***1R6F S2*** | ***cHTP*** | ***pHTP1*** | ***pHTP2*** | ***EVP-FB*** |  |
| ***1R6F S2*** | 0.17 |  |  |  |  |  |  | 0.21│0.26 |  |  |  |  |  |  |
| ***cHTP*** | 0.17│0.18 | 0.3│0.32 |  |  |  |  |  | 0.21│0.22 | 0.46│0.39 |  |  |  |  |  |
| ***pHTP1*** | 0.17│0.18 | 0.3│0.32 | 3.2 |  |  |  |  | 0.21│0.18 | 1.45│1.80 | 3.89│3.19 |  |  |  |  |
| ***pHTP2*** | 0.17│0.18 | 0.95│1.0 | ND | N/A |  |  |  | 0.21│0.25 | 1.45│1.40 | ND | ND |  |  |  |
| ***EVP-FB*** | 0.17│0.18 | 0.17│0.18 | 3.2 | 10 | ND |  |  | 0.38│0.29 | 0.26│0.29 | 0.69│0.52 | 3.19│2.93 | ND |  |  |
| ***EVP-NS*** | 0.17│0.18 | 0.95│1.0 | 1.8 | ND | ND | ND |  | 0.38│0.33 | 1.45│1.87 | 3.89│3.33 | ND | ND | ND |  |
| ***EVP-0*** | 0.17│0.18 | 0.17│0.18 | 3.2 | ND | ND | N/A | N/A |  |  |  |  |  |  |  |
| Value = Extract in Column 1 [%bPBS or µg/mL Nicotine]│Comparison Extract [%bPBS or µg/mL Nicotine]. If there is only one value, then the concentrations were the same in both samples.  **ND** = no statistically significant differences between samples at any concentration OR no consistent statistically significant differences to determine POD concentration.  **N/A** = dose-response curves were not statistically different (POD not applicable). | | | | | | | | | | | | | |  |

Supplemental Table S3: Viability/Arachidonic Acid Dose-Response Curve F Test Results (p-values)

| ***bPBS (%)*** | | | | | | | | ***Nicotine (µg/mL)*** | | | | | | |
| --- | --- | --- | --- | --- | --- | --- | --- | --- | --- | --- | --- | --- | --- | --- |
|  | ***1R6F S1*** | ***1R6F S2*** | ***cHTP*** | ***pHTP1*** | ***pHTP2*** | ***EVP-FB*** | ***EVP-NS*** | ***1R6F S1*** | ***1R6F S2*** | ***cHTP*** | ***pHTP1*** | ***pHTP2*** | ***EVP-FB*** |  |
| ***1R6F S2*** | <0.0001 |  |  |  |  |  |  | <0.0001 |  |  |  |  |  |  |
| ***cHTP*** | <0.0001 | <0.0001 |  |  |  |  |  | <0.0001 | <0.0001 |  |  |  |  |  |
| ***pHTP1*** | <0.0001 | <0.0001 | <0.0001 |  |  |  |  | <0.0001 | <0.0001 | 0.0019 |  |  |  |  |
| ***pHTP2*** | <0.0001 | <0.0001 | <0.0001 | 0.0727 |  |  |  | <0.0001 | <0.0001 | <0.0001 | 0.1216 |  |  |  |
| ***EVP-FB*** | <0.0001 | <0.0001 | <0.0001 | <0.0001 | <0.0001 |  |  | <0.0001 | <0.0001 | <0.0001 | <0.0001 | 0.0011 |  |  |
| ***EVP-NS*** | <0.0001 | <0.0001 | <0.0001 | <0.0001 | <0.0001 | 0.0004 |  | <0.0001 | <0.0001 | <0.0001 | 0.0234 | 0.0001 | <0.0001 |  |
| ***EVP-0*** | <0.0001 | <0.0001 | <0.0001 | <0.0001 | <0.0001 | <0.0001 | <0.0001 |  |  |  |  |  |  |  |

Treatments that did not have significantly different dose-response curves are highlighted in yellow (p<0.05).

Supplemental Table S4: Points of departure (POD): Viability/ Arachidonic Acid

| ***POD bPBS (%)*** | | | | | | | | ***POD Nicotine (µg/mL)*** | | | | | | |
| --- | --- | --- | --- | --- | --- | --- | --- | --- | --- | --- | --- | --- | --- | --- |
|  | ***1R6F S1*** | ***1R6F S2*** | ***cHTP*** | ***pHTP1*** | ***pHTP2*** | ***EVP-FB*** | ***EVP-NS*** | ***1R6F S1*** | ***1R6F S2*** | ***cHTP*** | ***pHTP1*** | ***pHTP2*** | ***EVP-FB*** |  |
| ***1R6F S2*** | 0.17 |  |  |  |  |  |  | 0.21│0.26 |  |  |  |  |  |  |
| ***cHTP*** | 0.17│0.18 | 3.0│3.2 |  |  |  |  |  | 0.21│0.22 | 4.60│3.89 |  |  |  |  |  |
| ***pHTP1*** | 0.17│0.18 | 1.7│1.8 | 10 |  |  |  |  | 0.21│0.18 | 2.59│3.19 | 12.3│10.1 |  |  |  |  |
| ***pHTP2*** | 0.17│0.18 | 3.0│3.2 | 5.6 | N/A |  |  |  | 0.21│0.25 | 4.60│4.43 | 6.92│7.87 | N/A |  |  |  |
| ***EVP-FB*** | 0.17│0.18 | 1.7│1.8 | 10 | ND | ND |  |  | 0.38│0.29 | 2.59│2.93 | 12.3│9.28 | ND | ND |  |  |
| ***EVP-NS*** | 0.17│0.18 | 3.0│3.2 | ND | ND | ND | ND |  | 0.38│0.33 | 4.60│5.91 | 6.92│5.91 | ND | 14.0│10.52 | ND |  |
| ***EVP-0*** | 0.95│1.0 | 1.7│1.8 | 0.18 | 5.6 | 5.6 | ND | ND |  |  |  |  |  |  |  |
| Value = Extract in Column 1 [%bPBS or µg/mL Nicotine]│Comparison Extract [%bPBS or µg/mL Nicotine]. If there is only one value, then the concentrations were the same in both samples.  **ND** = no statistically significant differences between samples at any concentration OR no consistent statistically significant differences to determine POD concentration.  **N/A** = dose-response curves were not statistically different (POD not applicable). | | | | | | | | | | | | | |  |

Supplemental Table S5: Thymidine Dose-Response Curve F Test Results (p-values)

| ***bPBS (%)*** | | | | | | | | ***Nicotine (µg/mL)*** | | | | | | |
| --- | --- | --- | --- | --- | --- | --- | --- | --- | --- | --- | --- | --- | --- | --- |
|  | ***1R6F S1*** | ***1R6F S2*** | ***cHTP*** | ***pHTP1*** | ***pHTP2*** | ***EVP-FB*** | ***EVP-NS*** | ***1R6F S1*** | ***1R6F S2*** | ***cHTP*** | ***pHTP1*** | ***pHTP2*** | ***EVP-FB*** |  |
| ***1R6F S2*** | <0.0001 |  |  |  |  |  |  | <0.0001 |  |  |  |  |  |  |
| ***cHTP*** | <0.0001 | <0.0001 |  |  |  |  |  | <0.0001 | <0.0001 |  |  |  |  |  |
| ***pHTP1*** | <0.0001 | <0.0001 | 0.0009 |  |  |  |  | <0.0001 | <0.0001 | 0.3222 |  |  |  |  |
| ***pHTP2*** | <0.0001 | <0.0001 | 0.0026 | 0.0337 |  |  |  | <0.0001 | <0.0001 | <0.0001 | 0.004 |  |  |  |
| ***EVP-FB*** | <0.0001 | <0.0001 | <0.0001 | 0.0002 | 0.0138 |  |  | <0.0001 | <0.0001 | <0.0001 | 0.0003 | 0.0146 |  |  |
| ***EVP-NS*** | <0.0001 | <0.0001 | <0.0001 | 0.0004 | 0.0052 | 0.0349 |  | <0.0001 | <0.0001 | <0.0001 | 0.0006 | 0.0054 | 0.0548 |  |
| ***EVP-0*** | <0.0001 | <0.0001 | <0.0001 | <0.0001 | 0.0015 | 0.0217 | 0.9746 |  |  |  |  |  |  |  |

Treatments that did not have significantly different dose-response curves are highlighted in yellow (p<0.05).

Supplemental Table S6: Points of departure: Thymidine

| ***POD bPBS (%)*** | | | | | | | | ***POD Nicotine (µg/mL)*** | | | | | | |
| --- | --- | --- | --- | --- | --- | --- | --- | --- | --- | --- | --- | --- | --- | --- |
|  | ***1R6F S1*** | ***1R6F S2*** | ***cHTP*** | ***pHTP1*** | ***pHTP2*** | ***EVP-FB*** | ***EVP-NS*** | ***1R6F S1*** | ***1R6F S2*** | ***cHTP*** | ***pHTP1*** | ***pHTP2*** | ***EVP-FB*** |  |
| ***1R6F S2*** | 1.7 |  |  |  |  |  |  | 2.14│2.59 |  |  |  |  |  |  |
| ***cHTP*** | 1.7│1.8 | 0.95│1.0 |  |  |  |  |  | 2.14│2.19 | 1.45│1.23 |  |  |  |  |  |
| ***pHTP1*** | 1.7│1.8 | 3.0│3.2 | ND |  |  |  |  | 2.14│1.80 | 2.59│3.19 | N/A |  |  |  |  |
| ***pHTP2*** | 0.95│1.0 | 0.95│1.0 | ND | ND |  |  |  | 1.20│1.40 | 1.45│1.40 | ND | 10.1│7.87 |  |  |  |
| ***EVP-FB*** | 1.7│1.8 | 0.95│1.0 | ND | 10 | ND |  |  | 2.14│1.65 | 1.45│1.65 | ND | 10.1│9.28 | ND |  |  |
| ***EVP-NS*** | 1.7│1.8 | 0.95│1.0 | ND | ND | ND | ND |  | 2.14│1.87 | 1.45│1.87 | ND | ND | ND | N/A |  |
| ***EVP-0*** | 1.7│1.8 | 0.95│1.0 | ND | 10 | ND | ND | N/A |  |  |  |  |  |  |  |
| Value = Extract in Column 1 [%bPBS or µg/mL Nicotine]│Comparison Extract [%bPBS or µg/mL Nicotine]. If there is only one value, then the concentrations were the same in both samples.  **ND** = no statistically significant differences between samples at any concentration OR no consistent statistically significant differences to determine POD concentration.  **N/A** = dose-response curves were not statistically different (POD not applicable). | | | | | | | | | | | | | |  |

Supplemental Table S7: 2’deoxycycidine Dose-Response Curve F Test Results (p-values)

| ***bPBS (%)*** | | | | | | | | ***Nicotine (µg/mL)*** | | | | | | |
| --- | --- | --- | --- | --- | --- | --- | --- | --- | --- | --- | --- | --- | --- | --- |
|  | ***1R6F S1*** | ***1R6F S2*** | ***cHTP*** | ***pHTP1*** | ***pHTP2*** | ***EVP-FB*** | ***EVP-NS*** | ***1R6F S1*** | ***1R6F S2*** | ***cHTP*** | ***pHTP1*** | ***pHTP2*** | ***EVP-FB*** |  |
| ***1R6F S2*** | <0.0001 |  |  |  |  |  |  | <0.0001 |  |  |  |  |  |  |
| ***cHTP*** | <0.0001 | <0.0001 |  |  |  |  |  | <0.0001 | <0.0001 |  |  |  |  |  |
| ***pHTP1*** | <0.0001 | <0.0001 | 0.0006 |  |  |  |  | <0.0001 | <0.0001 | 0.4233 |  |  |  |  |
| ***pHTP2*** | <0.0001 | <0.0001 | <0.0001 | 0.077 |  |  |  | <0.0001 | <0.0001 | <0.0001 | 0.0439 |  |  |  |
| ***EVP-FB*** | <0.0001 | <0.0001 | <0.0001 | 0.1372 | 0.188 |  |  | <0.0001 | <0.0001 | <0.0001 | 0.0084 | 0.0321 |  |  |
| ***EVP-NS*** | <0.0001 | <0.0001 | 0.0032 | 0.5514 | 0.84 | 0.7544 |  | <0.0001 | <0.0001 | <0.0001 | 0.4994 | 0.8654 | 0.3387 |  |
| ***EVP-0*** | <0.0001 | <0.0001 | <0.0001 | 0.1982 | 0.2686 | 0.5011 | 0.7353 |  |  |  |  |  |  |  |

Treatments that did not have significantly different dose-response curves are highlighted in yellow (p<0.05).

Supplemental Table S8: Points of departure: 2’deoxycycidine

| ***POD bPBS (%)*** | | | | | | | | ***POD Nicotine (µg/mL)*** | | | | | | |
| --- | --- | --- | --- | --- | --- | --- | --- | --- | --- | --- | --- | --- | --- | --- |
|  | ***1R6F S1*** | ***1R6F S2*** | ***cHTP*** | ***pHTP1*** | ***pHTP2*** | ***EVP-FB*** | ***EVP-NS*** | ***1R6F S1*** | ***1R6F S2*** | ***cHTP*** | ***pHTP1*** | ***pHTP2*** | ***EVP-FB*** |  |
| ***1R6F S2*** | 1.7 |  |  |  |  |  |  | 2.14│2.59 |  |  |  |  |  |  |
| ***cHTP*** | 0.95│1.0 | 1.7│1.8 |  |  |  |  |  | 1.20│1.23 | 2.59│2.19 |  |  |  |  |  |
| ***pHTP1*** | 0.95│1.0 | 0.3│0.32 | ND |  |  |  |  | 1.20│1.01 | 0.46│0.57 | N/A |  |  |  |  |
| ***pHTP2*** | 0.95│1.0 | 1.7│1.8 | ND | N/A |  |  |  | 1.20│1.40 | 2.59│2.49 | ND | ND |  |  |  |
| ***EVP-FB*** | 0.95│1.0 | 1.7│1.8 | 10 | N/A | N/A |  |  | 0.68│0.52 | 2.59│2.93 | 12.3│9.28 | ND | ND |  |  |
| ***EVP-NS*** | 0.95│1.0 | 1.7│1.8 | ND | N/A | N/A | N/A |  | 1.20│1.05 | 2.59│3.33 | 12.3│10.52 | N/A | N/A | N/A |  |
| ***EVP-0*** | 0.95│1.0 | 0.3│0.32 | 10 | N/A | N/A | N/A | N/A |  |  |  |  |  |  |  |
| Value = Extract in Column 1 [%bPBS or µg/mL Nicotine]│Comparison Extract [%bPBS or µg/mL Nicotine]. If there is only one value, then the concentrations were the same in both samples.  **ND** = no statistically significant differences between samples at any concentration OR no consistent statistically significant differences to determine POD concentration.  **N/A** = dose-response curves were not statistically different (POD not applicable). | | | | | | | | | | | | | |  |

Supplemental Table S9: Cell viability Dose-Response Curve F Test Results (p-values)

| ***bPBS (%)*** | | | | | | | | ***Nicotine (µg/mL)*** | | | | | | |
| --- | --- | --- | --- | --- | --- | --- | --- | --- | --- | --- | --- | --- | --- | --- |
|  | ***1R6F S1*** | ***1R6F S2*** | ***cHTP*** | ***pHTP1*** | ***pHTP2*** | ***EVP-FB*** | ***EVP-NS*** | ***1R6F S1*** | ***1R6F S2*** | ***cHTP*** | ***pHTP1*** | ***pHTP2*** | ***EVP-FB*** |  |
| ***1R6F S2*** | <0.0001 |  |  |  |  |  |  | <0.0001 |  |  |  |  |  |  |
| ***cHTP*** | <0.0001 | <0.0001 |  |  |  |  |  | <0.0001 | <0.0001 |  |  |  |  |  |
| ***pHTP1*** | <0.0001 | <0.0001 | <0.0001 |  |  |  |  | <0.0001 | <0.0001 | 0.0093 |  |  |  |  |
| ***pHTP2*** | <0.0001 | <0.0001 | <0.0001 | 0.0091 |  |  |  | <0.0001 | <0.0001 | <0.0001 | 0.0128 |  |  |  |
| ***EVP-FB*** | <0.0001 | <0.0001 | <0.0001 | 0.0974 | 0.9161 |  |  | <0.0001 | <0.0001 | <0.0001 | 0.1133 | 0.8912 |  |  |
| ***EVP-NS*** | <0.0001 | <0.0001 | <0.0001 | 0.0031 | 0.6802 | 0.3935 |  | <0.0001 | <0.0001 | <0.0001 | 0.0015 | 0.4061 | 0.2984 |  |
| ***EVP-0*** | <0.0001 | <0.0001 | <0.0001 | 0.0106 | 0.7495 | 0.437 | 0.3982 |  |  |  |  |  |  |  |

Treatments that did not have significantly different dose-response curves are highlighted in yellow (p<0.05).

Supplemental Table S10: Points of departure: Cell viability

| ***POD bPBS (%)*** | | | | | | | | ***POD Nicotine (µg/mL)*** | | | | | | |
| --- | --- | --- | --- | --- | --- | --- | --- | --- | --- | --- | --- | --- | --- | --- |
|  | ***1R6F S1*** | ***1R6F S2*** | ***cHTP*** | ***pHTP1*** | ***pHTP2*** | ***EVP-FB*** | ***EVP-NS*** | ***1R6F S1*** | ***1R6F S2*** | ***cHTP*** | ***pHTP1*** | ***pHTP2*** | ***EVP-FB*** |  |
| ***1R6F S2*** | 0.53 |  |  |  |  |  |  | 0.68│0.82 |  |  |  |  |  |  |
| ***cHTP*** | 0.95│1.0 | 3.0│3.2 |  |  |  |  |  | 1.20│1.23 | 4.60│3.89 |  |  |  |  |  |
| ***pHTP1*** | 0.17│0.18 | 3.0│3.2 | ND |  |  |  |  | 0.21│0.18 | 2.59│3.19 | ND |  |  |  |  |
| ***pHTP2*** | 0.95│1.0 | 3.0│3.2 | ND | 10 |  |  |  | 1.20│1.40 | 4.60│4.43 | ND | 10.1│7.87 |  |  |  |
| ***EVP-FB*** | 0.95│1.0 | 1.7│1.8 | ND | N/A | N/A |  |  | 0.38│0.29 | 2.59│2.93 | ND | N/A | N/A |  |  |
| ***EVP-NS*** | 0.95│1.0 | 1.7│1.8 | 10 | ND | N/A | N/A |  | 1.20│1.05 | 2.59│3.33 | 12.3│10.52 | ND | N/A | N/A |  |
| ***EVP-0*** | 0.95│1.0 | 3.0│3.2 | 5.6 | 10 | N/A | N/A | N/A |  |  |  |  |  |  |  |
| Value = Extract in Column 1 [%bPBS or µg/mL Nicotine]│Comparison Extract [%bPBS or µg/mL Nicotine]. If there is only one value, then the concentrations were the same in both samples.  **ND** = no statistically significant differences between samples at any concentration OR no consistent statistically significant differences to determine POD concentration.  **N/A** = dose-response curves were not statistically different (POD not applicable). | | | | | | | | | | | | | |  |

Supplemental Table S11: Lactic acid Dose-Response Curve F Test Results (p-values)

| ***bPBS (%)*** | | | | | | | | ***Nicotine (µg/mL)*** | | | | | | |
| --- | --- | --- | --- | --- | --- | --- | --- | --- | --- | --- | --- | --- | --- | --- |
|  | ***1R6F S1*** | ***1R6F S2*** | ***cHTP*** | ***pHTP1*** | ***pHTP2*** | ***EVP-FB*** | ***EVP-NS*** | ***1R6F S1*** | ***1R6F S2*** | ***cHTP*** | ***pHTP1*** | ***pHTP2*** | ***EVP-FB*** |  |
| ***1R6F S2*** | <0.0001 |  |  |  |  |  |  | <0.0001 |  |  |  |  |  |  |
| ***cHTP*** | <0.0001 | <0.0001 |  |  |  |  |  | <0.0001 | <0.0001 |  |  |  |  |  |
| ***pHTP1*** | <0.0001 | <0.0001 | <0.0001 |  |  |  |  | <0.0001 | <0.0001 | <0.0001 |  |  |  |  |
| ***pHTP2*** | <0.0001 | <0.0001 | <0.0001 | 0.3903 |  |  |  | <0.0001 | <0.0001 | <0.0001 | 0.014 |  |  |  |
| ***EVP-FB*** | <0.0001 | <0.0001 | <0.0001 | <0.0001 | <0.0001 |  |  | <0.0001 | <0.0001 | <0.0001 | <0.0001 | <0.0001 |  |  |
| ***EVP-NS*** | <0.0001 | 0.0028 | <0.0001 | 0.0002 | 0.1481 | 0.0018 |  | <0.0001 | <0.0001 | <0.0001 | <0.0001 | 0.0127 | 0.0061 |  |
| ***EVP-0*** | <0.0001 | 0.0013 | <0.0001 | <0.0001 | 0.0037 | 0.2003 | 0.194 |  |  |  |  |  |  |  |

Treatments that did not have significantly different dose-response curves are highlighted in yellow (p<0.05).

Supplemental Table S12: Points of departure: Lactic acid

| ***POD bPBS (%)*** | | | | | | | | ***POD Nicotine (µg/mL)*** | | | | | | |
| --- | --- | --- | --- | --- | --- | --- | --- | --- | --- | --- | --- | --- | --- | --- |
|  | ***1R6F S1*** | ***1R6F S2*** | ***cHTP*** | ***pHTP1*** | ***pHTP2*** | ***EVP-FB*** | ***EVP-NS*** | ***1R6F S1*** | ***1R6F S2*** | ***cHTP*** | ***pHTP1*** | ***pHTP2*** | ***EVP-FB*** |  |
| ***1R6F S2*** | 0.17 |  |  |  |  |  |  | 0.21│0.26 |  |  |  |  |  |  |
| ***cHTP*** | 0.17│0.18 | 0.17│0.18 |  |  |  |  |  | 0.21│0.22 | 0.26│0.22 |  |  |  |  |  |
| ***pHTP1*** | 0.17│0.18 | 0.3│0.32 | ND |  |  |  |  | 0.21│0.18 | 1.45│1.80 | ND |  |  |  |  |
| ***pHTP2*** | 0.17│0.18 | 0.17│0.18 | ND | N/A |  |  |  | 0.21│0.25 | 0.26│0.25 | ND | 10.1│7.87 |  |  |  |
| ***EVP-FB*** | 0.17│0.18 | 0.17│0.18 | ND | 10 | 10 |  |  | 0.38│0.29 | 0.26│0.29 | 1.23│0.93 | 1.01│0.93 | 14.0│16.51 |  |  |
| ***EVP-NS*** | 0.17│0.18 | 0.17│0.18 | ND | ND | N/A | ND |  | 0.38│0.33 | 0.26│0.33 | 2.19│1.87 | 10.1│10.52 | 14.0│10.52 | ND |  |
| ***EVP-0*** | 0.17│0.18 | 0.17│0.18 | ND | 10 | 10 | N/A | N/A |  |  |  |  |  |  |  |
| Value = Extract in Column 1 [%bPBS or µg/mL Nicotine]│Comparison Extract [%bPBS or µg/mL Nicotine]. If there is only one value, then the concentrations were the same in both samples.  **ND** = no statistically significant differences between samples at any concentration OR no consistent statistically significant differences to determine POD concentration.  **N/A** = dose-response curves were not statistically different (POD not applicable). | | | | | | | | | | | | | |  |

Supplemental Table S13: Arachidonic acid Dose-Response Curve F Test Results (p-values)

| ***bPBS (%)*** | | | | | | | | ***Nicotine (µg/mL)*** | | | | | | |
| --- | --- | --- | --- | --- | --- | --- | --- | --- | --- | --- | --- | --- | --- | --- |
|  | ***1R6F S1*** | ***1R6F S2*** | ***cHTP*** | ***pHTP1*** | ***pHTP2*** | ***EVP-FB*** | ***EVP-NS*** | ***1R6F S1*** | ***1R6F S2*** | ***cHTP*** | ***pHTP1*** | ***pHTP2*** | ***EVP-FB*** |  |
| ***1R6F S2*** | 0.0002 |  |  |  |  |  |  | 0.0016 |  |  |  |  |  |  |
| ***cHTP*** | 0.0003 | <0.0001 |  |  |  |  |  | 0.0003 | <0.0001 |  |  |  |  |  |
| ***pHTP1*** | <0.0001 | <0.0001 | <0.0001 |  |  |  |  | <0.0001 | <0.0001 | <0.0001 |  |  |  |  |
| ***pHTP2*** | 0.0001 | <0.0001 | <0.0001 | <0.0001 |  |  |  | 0.0001 | <0.0001 | <0.0001 | <0.0001 |  |  |  |
| ***EVP-FB*** | <0.0001 | <0.0001 | <0.0001 | <0.0001 | <0.0001 |  |  | <0.0001 | <0.0001 | <0.0001 | <0.0001 | <0.0001 |  |  |
| ***EVP-NS*** | <0.0001 | <0.0001 | <0.0001 | <0.0001 | <0.0001 | <0.0001 |  | <0.0001 | <0.0001 | <0.0001 | <0.0001 | <0.0001 | <0.0001 |  |
| ***EVP-0*** | <0.0001 | <0.0001 | <0.0001 | <0.0001 | <0.0001 | 0.0003 | <0.0001 |  |  |  |  |  |  |  |

Treatments that did not have significantly different dose-response curves are highlighted in yellow (p<0.05).

Supplemental Table S14: Points of departure: Arachidonic acid

| ***POD bPBS (%)*** | | | | | | | | ***POD Nicotine (µg/mL)*** | | | | | | |
| --- | --- | --- | --- | --- | --- | --- | --- | --- | --- | --- | --- | --- | --- | --- |
|  | ***1R6F S1*** | ***1R6F S2*** | ***cHTP*** | ***pHTP1*** | ***pHTP2*** | ***EVP-FB*** | ***EVP-NS*** | ***1R6F S1*** | ***1R6F S2*** | ***cHTP*** | ***pHTP1*** | ***pHTP2*** | ***EVP-FB*** |  |
| ***1R6F S2*** | ND |  |  |  |  |  |  | ND |  |  |  |  |  |  |
| ***cHTP*** | ND | 0.17│0.18 |  |  |  |  |  | ND | 0.26│0.22 |  |  |  |  |  |
| ***pHTP1*** | ND | 1.7│1.8 | 0.18 |  |  |  |  | ND | 4.60│5.68 | 0.22│0.18 |  |  |  |  |
| ***pHTP2*** | ND | 3.0│3.2 | 10 | 10 |  |  |  | ND | 4.68│4.43 | 12.3│14.0 | 10.1│7.87 |  |  |  |
| ***EVP-FB*** | 0.17│0.18 | 3.0│3.2 | 0.18 | 5.6 | 1.8 |  |  | ND | 4.60│5.22 | 0.39│0.29 | 5.68│5.22 | 2.49│2.93 |  |  |
| ***EVP-NS*** | ND | 3.0│3.2 | ND | 5.6 | 5.6 | 0.18 |  | ND | 4.60│5.91 | 12.3│10.52 | 10.1│10.52 | 14.0│10.52 | 0.29│0.33 |  |
| ***EVP-0*** | ND | 1.7│1.8 | 0.18 | 5.6 | 5.6 | ND | 0.18 |  |  |  |  |  |  |  |
| Value = Extract in Column 1 [%bPBS or µg/mL Nicotine]│Comparison Extract [%bPBS or µg/mL Nicotine]. If there is only one value, then the concentrations were the same in both samples.  **ND** = no statistically significant differences between samples at any concentration OR no consistent statistically significant differences to determine POD concentration.  **N/A** = dose-response curves were not statistically different (POD not applicable). | | | | | | | | | | | | | |  |


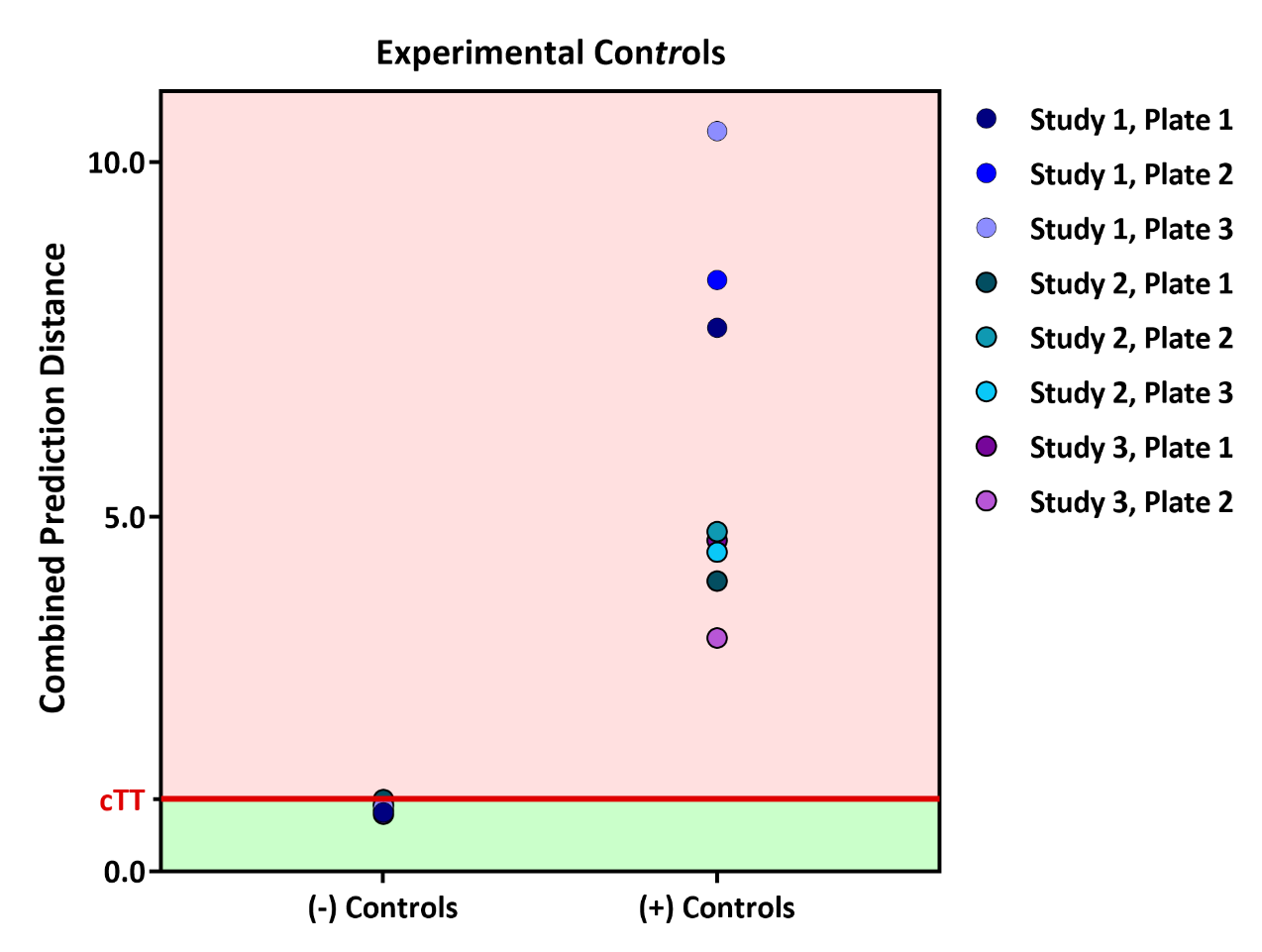


Supplementary Figure S4: Prediction Model Results for Negative and Positive Controls on Each Plate.

*Controls were included on each cell culture plate and consisted of cells treated with Verapamil at concentrations of 0.005 μM (negative control) and 30 μM (positive control).* *Verapamil was selected for the assay controls because it causes changes in all four biomarkers. The most sensitive biomarker (which is used for the CPD determination) is viability/lactic acid, so the presented CPD values are from the viability/lactic acid prediction distance. The actual response for viability/lactic acid ranges from 0.03 – 0.04 in Study 1, 0.07 - 0.9 for Study 2, and 0.07 – 0.10 for Study 3. The prediction distance for these values ranges from 3.3 – 10.4.*

| Supplemental Table S15: Individual and Mean Endpoint Values for (-) – Nicotine (0.01 – 30 µg/mL) | | | | | | | | | | | | | | | | |
| --- | --- | --- | --- | --- | --- | --- | --- | --- | --- | --- | --- | --- | --- | --- | --- | --- |
| **Endpoint 🡪** | **Cell Viability** | | | | **Lactic Acid** | | | | **Viability/Lactic Acid** | | | | **Arachidonic Acid** | | | |
| **[Nicotine] (µg/mL)** | **Rep 1** | **Rep 2** | **Rep 3** | **Mean (±SEM)** | **Rep 1** | **Rep 2** | **Rep 3** | **Mean (±SEM)** | **Rep 1** | **Rep 2** | **Rep 3** | **Mean (±SEM)** | **Rep 1** | **Rep 2** | **Rep 3** | **Mean (±SEM)** |
| 0.01 | 0.88 | 0.98 | 1.05 | 0.97 (±0.05) | 1.00 | 1.23 | 1.07 | 1.10 (±0.07) | 0.88 | 0.80 | 0.98 | 0.89 (±0.05) | 1.09 | 1.06 | 1.04 | 1.06 (±0.01) |
| 0.03 | 0.89 | 1.03 | 1.06 | 1.00 (±0.05) | 0.92 | 0.96 | 1.20 | 1.03 (±0.09) | 0.96 | 1.08 | 0.88 | 0.97 (±0.06) | 1.09 | 1.06 | 1.07 | 1.07 (±0.01) |
| 0.1 | 0.90 | 1.00 | 1.07 | 0.99 (±0.05) | 0.98 | 1.08 | 1.13 | 1.06 (±0.04) | 0.92 | 0.93 | 0.95 | 0.93 (±0.01) | 1.08 | N/A | 1.08 | 1.08 (±0.00) |
| 0.3 | 0.95 | 0.96 | 1.09 | 1.00 (±0.04) | 1.07 | 1.07 | 1.03 | 1.06 (±0.01) | 0.89 | 0.89 | 1.05 | 0.94 (±0.05) | 1.12 | 1.09 | 1.04 | 1.08 (±0.02) |
| 1 | 0.97 | 0.96 | N/A | 0.97 (±0.00) | 0.93 | N/A | 1.00 | 0.97 (±0.04) | 1.05 | N/A | N/A | 1.05 (±0.00) | 1.08 | 1.10 | 1.05 | 1.08 (±0.01) |
| 3 | 1.00 | 1.01 | 1.09 | 1.03 (±0.03) | 1.04 | 0.95 | 1.03 | 1.01 (±0.03) | 0.96 | 1.06 | 1.06 | 1.03 (±0.03) | 1.11 | 1.09 | 1.06 | 1.09 (±0.01) |
| 10 | 1.06 | 1.06 | 1.11 | 1.08 (±0.02) | 1.04 | 1.08 | 1.18 | 1.10 (±0.04) | 1.02 | 0.99 | 0.93 | 0.98 (±0.02) | 1.09 | 1.08 | 1.09 | 1.09 (±0.00) |
| 30 | 1.05 | 1.04 | 1.08 | 1.06 (±0.01) | 1.36 | 1.19 | 1.32 | 1.29 (±0.05) | 0.77 | 0.87 | 0.82 | 0.82 (±0.03) | 1.12 | 1.07 | 1.07 | 1.09 (±0.01) |
| **Endpoint 🡪** | **Viability/Arachidonic Acid** | | | | **Thymidine** | | | | **2’-Deoxycytidine** | | | | **CPD** | |  |  |
| **[Nicotine] (µg/mL)** | **Rep 1** | **Rep 2** | **Rep 3** | **Mean (±SEM)** | **Rep 1** | **Rep 2** | **Rep 3** | **Mean (±SEM)** | **Rep 1** | **Rep 2** | **Rep 3** | **Mean (±SEM)** |  |  |  |  |
| 0.01 | 0.81 | 0.93 | 1.01 | 0.92 (±0.06) | 1.01 | 0.86 | 1.08 | 0.98 (±0.07) | N/A | 1.09 | 1.05 | 1.07 (±0.02) | 0.851 | |  |  |
| 0.03 | 0.81 | 0.98 | 0.99 | 0.93 (±0.06) | 0.77 | 0.94 | 0.97 | 0.89 (±0.06) | 1.07 | N/A | 1.01 | 1.04 (±0.03) | 0.952 | |  |  |
| 0.1 | 0.83 | N/A | 0.99 | 0.91 (±0.08) | N/A | 1.08 | 1.00 | 1.04 (±0.04) | 1.09 | N/A | 1.00 | 1.05 (±0.04) | 0.867 | |  |  |
| 0.3 | 0.85 | 0.88 | 1.04 | 0.93 (±0.06) | 0.71 | 0.96 | 0.99 | 0.89 (±0.09) | 0.96 | N/A | 0.98 | 0.97 (±0.01) | 0.928 | |  |  |
| 1 | 0.90 | 0.88 | N/A | 0.89 (±0.01) | 0.98 | 1.02 | 1.03 | 1.01 (±0.02) | N/A | 1.01 | 1.02 | 1.02 (±0.00) | 0.806 | |  |  |
| 3 | 0.90 | 0.92 | 1.03 | 0.95 (±0.04) | 1.00 | 1.12 | 1.01 | 1.05 (±0.04) | 0.98 | 0.98 | 1.02 | 0.99 (±0.01) | 0.885 | |  |  |
| 10 | 0.97 | 0.99 | 1.01 | 0.99 (±0.01) | 0.96 | 1.07 | 1.03 | 1.02 (±0.03) | 0.94 | 1.07 | 1.12 | 1.04 (±0.05) | 0.849 | |  |  |
| 30 | 0.12 | 0.06 | 0.07 | 0.08 (±0.02) | 0.00 | 0.00 | 0.00 | 0 (±0) | 0.00 | 0.00 | 0.00 | 0 (±0) | 10.081 | |  |  |
| Values have been normalized to internal standards and reference controls  **CPD**: Combined Prediction Distance. **N/A**: Outlier value that was removed from analysis (determined by Grubbs’ test). | | | | | | | | | | | | | | | | |

| **Supplemental Table S16: Individual and Mean Endpoint Values for (-) – Nicotine (18 - 1000 µg/mL)** | | | | | | | | | | | | | | | | |
| --- | --- | --- | --- | --- | --- | --- | --- | --- | --- | --- | --- | --- | --- | --- | --- | --- |
| **Endpoint 🡪** | **Cell Viability** | | | | **Lactic Acid** | | | | **Viability/Lactic Acid** | | | | **Arachidonic Acid** | | | |
| **[Nicotine] (µg/mL)** | **Rep 1** | **Rep 2** | **Rep 3** | **Mean (±SEM)** | **Rep 1** | **Rep 2** | **Rep 3** | **Mean (±SEM)** | **Rep 1** | **Rep 2** | **Rep 3** | **Mean (±SEM)** | **Rep 1** | **Rep 2** | **Rep 3** | **Mean (±SEM)** |
| 18 | 1.13 | 1.03 | 0.96 | 1.04 (±0.05) | 1.46 | 1.22 | 1.30 | 1.33 (±0.07) | 0.78 | 0.84 | 0.73 | 0.78 (±0.03) | 0.91 | 0.96 | 0.98 | 0.95 (±0.02) |
| 32 | N/A | 1.01 | 1.02 | 1.02 (±0.00) | 1.68 | 1.40 | 1.52 | 1.53 (±0.08) | N/A | 0.73 | 0.67 | 0.70 (±0.03) | 0.92 | 0.99 | 0.98 | 0.96 (±0.02) |
| 56 | 1.10 | 1.02 | 1.09 | 1.07 (±0.03) | 1.77 | 1.64 | 1.74 | 1.72 (±0.04) | 0.62 | 0.62 | 0.63 | 0.62 (±0.00) | 0.88 | 0.99 | 0.94 | 0.93 (±0.03) |
| 100 | 1.06 | 1.02 | 1.03 | 1.04 (±0.01) | 2.32 | 1.95 | 2.11 | 2.13 (±0.11) | 0.46 | 0.52 | 0.49 | 0.49 (±0.02) | 0.70 | 0.76 | 0.80 | 0.75 (±0.03) |
| 178 | 0.88 | 0.86 | 0.83 | 0.85 (±0.01) | 2.18 | 2.17 | 2.19 | 2.18 (±0.01) | 0.40 | 0.39 | 0.38 | 0.39 (±0.01) | 0.32 | 0.32 | 0.34 | 0.32 (±0.01) |
| 316 | 0.91 | 0.82 | 0.84 | 0.86 (±0.03) | 1.66 | N/A | 1.69 | 1.68 (±0.02) | 0.55 | N/A | 0.50 | 0.52 (±0.03) | 0.40 | 0.40 | 0.42 | 0.41 (±0.01) |
| 562 | 0.58 | 0.55 | 0.57 | 0.56 (±0.01) | 2.52 | 2.63 | 2.62 | 2.59 (±0.03) | 0.23 | 0.21 | 0.22 | 0.22 (±0.01) | 1.35 | 1.67 | 1.52 | 1.51 (±0.09) |
| 1,000 | 0.16 | 0.18 | 0.18 | 0.17 (±0.01) | 3.32 | 3.29 | 3.54 | 3.38 (±0.08) | 0.05 | 0.06 | 0.05 | 0.05 (±0.00) | 3.35 | 3.47 | 3.45 | 3.42 (±0.04) |
| **Endpoint 🡪** | **Viability/Arachidonic Acid** | | | | **Thymidine** | | | | **2’-Deoxycytidine** | | | | **CPD** | |  |  |
| **[Nicotine] (µg/mL)** | **Rep 1** | **Rep 2** | **Rep 3** | **Mean (±SEM)** | **Rep 1** | **Rep 2** | **Rep 3** | **Mean (±SEM)** | **Rep 1** | **Rep 2** | **Rep 3** | **Mean (±SEM)** |  |  |  |  |
| 18 | 1.24 | 1.07 | 0.98 | 1.10 (±0.08) | 1.02 | 1.03 | 1.03 | 1.03 (±0.00) | 1.08 | 1.17 | 1.10 | 1.11 (±0.03) | 0.874 | |  |  |
| 32 | N/A | 1.03 | 1.04 | 1.03 (±0.00) | 1.07 | 1.03 | 1.01 | 1.04 (±0.02) | 1.10 | 1.16 | 1.17 | 1.14 (±0.02) | 0.919 | |  |  |
| 56 | 1.25 | 1.03 | 1.17 | 1.15 (±0.06) | 1.09 | 1.12 | 1.11 | 1.11 (±0.01) | 1.21 | 1.23 | 1.32 | 1.25 (±0.04) | 0.975 | |  |  |
| 100 | 1.52 | 1.35 | 1.29 | 1.38 (±0.07) | 1.11 | 1.04 | 1.07 | 1.07 (±0.02) | 1.25 | 1.39 | 1.38 | 1.34 (±0.04) | 1.098 | |  |  |
| 178 | 2.77 | 2.70 | 2.45 | 2.64 (±0.10) | 1.23 | 1.12 | 1.22 | 1.19 (±0.04) | 1.27 | 1.34 | 1.38 | 1.33 (±0.03) | 1.569 | |  |  |
| 316 | 2.25 | 2.03 | 2.01 | 2.10 (±0.08) | 1.05 | N/A | 1.05 | 1.05 (±0.00) | 0.78 | 0.73 | 0.76 | 0.76 (±0.01) | 1.182 | |  |  |
| 562 | 0.43 | 0.33 | 0.38 | 0.38 (±0.03) | 0.49 | 0.53 | 0.51 | 0.51 (±0.01) | 0.20 | 0.26 | 0.23 | 0.23 (±0.01) | 3.741 | |  |  |
| 1,000 | 0.05 | 0.05 | 0.05 | 0.05 (±0.00) | 0.00 | 0.00 | 0.00 | 0 (±0) | 0.00 | 0.00 | 0.00 | 0 (±0) | 13.278 | |  |  |
| Values have been normalized to internal standards and reference controls  **CPD**: Combined Prediction Distance. **N/A**: Outlier value that was removed from analysis (determined by Grubbs’ test). | | | | | | | | | | | | | | |  |  |

| **Supplemental Table S17: Individual and Mean Endpoint Values for 1R6F S1** | | | | | | | | | | | | | | | | |
| --- | --- | --- | --- | --- | --- | --- | --- | --- | --- | --- | --- | --- | --- | --- | --- | --- |
| **Endpoint 🡪** | **Cell Viability** | | | | **Lactic Acid** | | | | **Viability/Lactic Acid** | | | | **Arachidonic Acid** | | | |
| **[1R6F S1]** | **Rep 1** | **Rep 2** | **Rep 3** | **Mean (±SEM)** | **Rep 1** | **Rep 2** | **Rep 3** | **Mean (±SEM)** | **Rep 1** | **Rep 2** | **Rep 3** | **Mean (±SEM)** | **Rep 1** | **Rep 2** | **Rep 3** | **Mean (±SEM)** |
| 0.053% | 1.07 | 1.04 | 1.08 | 1.06 (±0.011) | 0.94 | 0.93 | 1.05 | 0.97 (±0.041) | 1.14 | 1.13 | 1.03 | 1.10 (±0.035) | 1.06 | 1.05 | 1.07 | 1.06 (±0.005) |
| 0.095% | 1.03 | 1.11 | 0.95 | 1.03 (±0.048) | 0.90 | 0.91 | 0.92 | 0.91 (±0.008) | 1.15 | 1.22 | 1.03 | 1.13 (±0.057) | 1.03 | 1.03 | 1.02 | 1.03 (±0.003) |
| 0.17% | 1.07 | 1.01 | 1.03 | 1.04 (±0.019) | 0.77 | 0.69 | 0.84 | 0.77 (±0.046) | 1.39 | 1.47 | 1.22 | 1.36 (±0.072) | 0.99 | 0.95 | 1.00 | 0.98 (±0.016) |
| 0.30% | 1.09 | 1.05 | 1.07 | 1.07 (±0.011) | 0.72 | 0.72 | 0.78 | 0.74 (±0.021) | 1.53 | 1.46 | 1.37 | 1.45 (±0.046) | 0.90 | 0.97 | 0.98 | 0.95 (±0.027) |
| 0.53% | 1.06 | 1.06 | N/A | 1.06 (±0.000) | 0.71 | 0.52 | 0.64 | 0.63 (±0.055) | 1.49 | 2.03 | N/A | 1.76 (±0.268) | 0.95 | 0.95 | 0.98 | 0.96 (±0.012) |
| 0.95% | 0.63 | 0.82 | 0.75 | 0.73 (±0.055) | 1.94 | 0.91 | 0.85 | 1.23 (±0.355) | 0.33 | 0.91 | 0.88 | 0.70 (±0.189) | 1.47 | 1.17 | 1.17 | 1.27 (±0.098) |
| 1.7% | N/A | 0.00 | 0.00 | 0.00 (±0.000) | 1.97 | 2.09 | 2.04 | 2.04 (±0.033) | N/A | 0.00 | 0.00 | 0.00 (±0.000) | 1.27 | 1.25 | 1.22 | 1.25 (±0.015) |
| 3.0% | 0.00 | 0.02 | 0.00 | 0.01 (±0.007) | 2.16 | 1.90 | 2.06 | 2.04 (±0.074) | 0.00 | 0.01 | 0.00 | 0.00 (±0.003) | 1.14 | 1.74 | 1.22 | 1.37 (±0.190) |
| **Endpoint 🡪** | **Viability/Arachidonic Acid** | | | | **Thymidine** | | | | **2’-Deoxycytidine** | | | | **CPD** | |  |  |
| **[1R6F S1]** | **Rep 1** | **Rep 2** | **Rep 3** | **Mean (±SEM)** | **Rep 1** | **Rep 2** | **Rep 3** | **Mean (±SEM)** | **Rep 1** | **Rep 2** | **Rep 3** | **Mean (±SEM)** |  |  |  |  |
| 0.053% | 1.01 | 0.99 | 1.01 | 1.00 (±0.006) | 1.04 | 1.08 | 1.12 | 1.08 (±0.023) | 1.02 | 1.08 | 1.08 | 1.06 (±0.019) | 0.854 | |  |  |
| 0.095% | 1.00 | 1.08 | 0.93 | 1.00 (±0.044) | 1.01 | 1.15 | 1.04 | 1.07 (±0.041) | 1.08 | 1.03 | 1.01 | 1.04 (±0.021) | 0.814 | |  |  |
| 0.17% | 1.08 | 1.06 | 1.03 | 1.06 (±0.016) | 1.10 | 1.10 | 1.02 | 1.07 (±0.025) | 1.01 | 1.00 | 0.98 | 1.00 (±0.010) | 0.963 | |  |  |
| 0.30% | 1.22 | 1.08 | 1.09 | 1.13 (±0.045) | 1.07 | 1.06 | 1.08 | 1.07 (±0.007) | 0.96 | 1.01 | 1.09 | 1.02 (±0.036) | 1.015 | |  |  |
| 0.53% | 1.12 | 1.11 | N/A | 1.11 (±0.000) | 1.09 | 1.07 | 1.08 | 1.08 (±0.008) | 1.07 | N/A | 1.05 | 1.06 (±0.005) | 1.223 | |  |  |
| 0.95% | 0.43 | 0.70 | 0.64 | 0.59 (±0.081) | 0.71 | 0.87 | 0.94 | 0.84 (±0.066) | 0.48 | 0.70 | 0.70 | 0.63 (±0.074) | 1.238 | |  |  |
| 1.7% | 0.00 | 0.00 | 0.00 | 0.00 (±0.000) | 0.00 | 0.00 | 0.00 | 0.00 (±0.000) | 0.00 | 0.00 | 0.00 | 0.00 (±0.000) | Inf | |  |  |
| 3.0% | 0.00 | 0.01 | 0.00 | 0.00 (±0.004) | 0.00 | 0.00 | 0.00 | 0.00 (±0.000) | 0.00 | 0.00 | 0.00 | 0.00 (±0.000) | Inf | |  |  |
| Values have been normalized to internal standards and reference controls  **CPD**: Combined Prediction Distance. **N/A**: Outlier value that was removed from analysis (determined by Grubbs’ test). | | | | | | | | | | | | | | |  |  |

| **Supplemental Table S18: Individual and Mean Endpoint Values for 1R6F S2** | | | | | | | | | | | | | | | | |
| --- | --- | --- | --- | --- | --- | --- | --- | --- | --- | --- | --- | --- | --- | --- | --- | --- |
| **Endpoint 🡪** | **Cell Viability** | | | | **Lactic Acid** | | | | **Viability/Lactic Acid** | | | | **Arachidonic Acid** | | | |
| **[1R6F S2]** | **Rep 1** | **Rep 2** | **Rep 3** | **Mean (±SEM)** | **Rep 1** | **Rep 2** | **Rep 3** | **Mean (±SEM)** | **Rep 1** | **Rep 2** | **Rep 3** | **Mean (±SEM)** | **Rep 1** | **Rep 2** | **Rep 3** | **Mean (±SEM)** |
| 0.053% | 1.00 | 0.99 | 1.02 | 1.01 (±0.01) | 1.02 | 1.08 | 0.93 | 1.01 (±0.04) | 0.98 | 0.92 | 1.10 | 1.00 (±0.05) | 1.00 | 0.98 | 1.02 | 1.00 (±0.01) |
| 0.095% | 1.00 | 0.96 | 1.01 | 0.99 (±0.02) | 0.94 | 0.95 | 1.02 | 0.97 (±0.03) | 1.06 | 1.02 | 0.99 | 1.02 (±0.02) | 1.06 | 1.04 | 1.05 | 1.05 (±0.01) |
| 0.17% | 1.00 | 0.94 | 0.97 | 0.97 (±0.02) | 0.97 | 1.00 | 0.94 | 0.97 (±0.02) | 1.03 | 0.94 | 1.03 | 1.00 (±0.03) | 1.02 | 1.06 | 1.08 | 1.05 (±0.02) |
| 0.30% | 1.08 | 0.95 | 0.97 | 1.00 (±0.04) | 0.90 | 0.83 | 0.84 | 0.86 (±0.02) | 1.20 | 1.15 | 1.14 | 1.16 (±0.02) | 1.01 | 1.03 | 1.04 | 1.03 (±0.01) |
| 0.53% | 0.99 | 0.88 | 0.94 | 0.94 (±0.03) | 0.82 | 0.72 | 0.86 | 0.80 (±0.04) | 1.21 | 1.22 | 1.10 | 1.18 (±0.04) | 1.06 | 1.05 | 1.09 | 1.06 (±0.01) |
| 0.95% | 1.02 | 0.96 | 0.95 | 0.98 (±0.02) | 0.63 | 0.69 | 0.67 | 0.66 (±0.02) | 1.63 | 1.40 | 1.42 | 1.48 (±0.07) | 1.07 | 1.04 | 1.02 | 1.04 (±0.01) |
| 1.7% | N/A | 0.86 | 0.86 | 0.86 (±0.00) | 0.56 | 0.64 | 0.60 | 0.60 (±0.02) | N/A | 1.34 | 1.44 | 1.39 (±0.05) | N/A | 1.12 | 1.12 | 1.12 (±0.00) |
| 3.0% | 0.19 | 0.09 | 0.12 | 0.13 (±0.03) | 4.43 | 3.18 | 3.66 | 3.75 (±0.36) | 0.04 | 0.03 | 0.03 | 0.03 (±0.00) | 1.62 | 1.64 | 1.73 | 1.66 (±0.03) |
| **Endpoint 🡪** | **Viability/Arachidonic Acid** | | | | **Thymidine** | | | | **2’-Deoxycytidine** | | | | **CPD** | |  |  |
| **[1R6F S2]** | **Rep 1** | **Rep 2** | **Rep 3** | **Mean (±SEM)** | **Rep 1** | **Rep 2** | **Rep 3** | **Mean (±SEM)** | **Rep 1** | **Rep 2** | **Rep 3** | **Mean (±SEM)** |  |  |  |  |
| 0.053% | 1.01 | 1.01 | 1.00 | 1.01 (±0.00) | 0.92 | 0.93 | 0.93 | 0.92 (±0.00) | 0.96 | 1.10 | 0.94 | 1.00 (±0.05) | 0.967 | |  |  |
| 0.095% | 0.95 | 0.93 | 0.97 | 0.95 (±0.01) | 0.85 | 0.92 | 1.06 | 0.95 (±0.06) | 1.06 | 1.05 | 0.96 | 1.02 (±0.03) | 0.970 | |  |  |
| 0.17% | 0.98 | 0.89 | 0.90 | 0.92 (±0.03) | 0.94 | 0.90 | 0.96 | 0.93 (±0.02) | 0.83 | 1.05 | 0.89 | 0.92 (±0.07) | 0.971 | |  |  |
| 0.30% | 1.07 | 0.92 | 0.92 | 0.97 (±0.05) | 1.01 | 0.89 | 1.00 | 0.97 (±0.04) | 0.93 | 0.94 | 0.96 | 0.94 (±0.01) | 0.920 | |  |  |
| 0.53% | 0.93 | 0.84 | 0.87 | 0.88 (±0.03) | 0.92 | 0.92 | 1.05 | 0.96 (±0.04) | 0.88 | 0.71 | 0.91 | 0.83 (±0.06) | 0.978 | |  |  |
| 0.95% | 0.96 | 0.92 | 0.94 | 0.94 (±0.01) | 0.85 | 0.94 | 0.84 | 0.88 (±0.03) | 0.92 | 0.94 | 0.92 | 0.92 (±0.01) | 1.053 | |  |  |
| 1.7% | N/A | 0.77 | 0.77 | 0.77 (±0.00) | 0.90 | 0.77 | 0.88 | 0.85 (±0.04) | 0.77 | 0.75 | 0.79 | 0.77 (±0.01) | 1.124 | |  |  |
| 3.0% | 0.12 | 0.06 | 0.07 | 0.08 (±0.02) | 0.00 | 0.00 | 0.00 | 0 (±0) | 0.00 | 0.00 | 0.00 | 0 (±0) | 10.081 | |  |  |
| Values have been normalized to internal standards and reference controls  **CPD**: Combined Prediction Distance. **N/A**: Outlier value that was removed from analysis (determined by Grubbs’ test). | | | | | | | | | | | | | | |  |  |

| **Supplemental Table S19: Individual and Mean Endpoint Values for EVP-0** | | | | | | | | | | | | | | | | |
| --- | --- | --- | --- | --- | --- | --- | --- | --- | --- | --- | --- | --- | --- | --- | --- | --- |
| **Endpoint 🡪** | **Cell Viability** | | | | **Lactic Acid** | | | | **Viability/Lactic Acid** | | | | **Arachidonic Acid** | | | |
| **[EVP-0]** | **Rep 1** | **Rep 2** | **Rep 3** | **Mean (±SEM)** | **Rep 1** | **Rep 2** | **Rep 3** | **Mean (±SEM)** | **Rep 1** | **Rep 2** | **Rep 3** | **Mean (±SEM)** | **Rep 1** | **Rep 2** | **Rep 3** | **Mean (±SEM)** |
| 0.18% | 0.93 | 0.97 | 1.06 | 0.99 (±0.039) | 1.03 | 1.07 | 1.25 | 1.12 (±0.066) | 0.90 | 0.91 | 0.85 | 0.89 (±0.018) | 0.97 | 1.01 | 1.07 | 1.02 (±0.030) |
| 0.32% | 0.95 | 1.07 | 1.08 | 1.04 (±0.041) | N/A | 1.16 | 1.14 | 1.15 (±0.009) | N/A | 0.93 | 0.95 | 0.94 (±0.013) | 0.92 | 1.04 | 1.03 | 1.00 (±0.039) |
| 0.56% | 0.97 | 1.08 | 0.95 | 1.00 (±0.040) | 0.98 | 1.04 | 1.08 | 1.03 (±0.029) | 0.99 | 1.04 | 0.88 | 0.97 (±0.046) | 0.99 | 1.00 | 1.04 | 1.01 (±0.017) |
| 1.0% | 0.96 | 0.97 | 1.05 | 0.99 (±0.029) | 1.03 | 1.12 | 0.99 | 1.05 (±0.038) | 0.93 | 0.87 | 1.06 | 0.95 (±0.057) | 1.02 | 1.02 | 1.04 | 1.03 (±0.005) |
| 1.8% | 1.00 | 0.96 | 1.04 | 1.00 (±0.024) | 0.92 | 1.01 | 0.97 | 0.97 (±0.027) | 1.09 | 0.95 | 1.07 | 1.04 (±0.045) | 0.97 | 1.00 | 1.01 | 1.00 (±0.014) |
| 3.2% | 0.95 | 0.90 | 0.99 | 0.94 (±0.024) | 0.90 | 1.01 | 0.87 | 0.93 (±0.045) | 1.05 | 0.89 | 1.14 | 1.03 (±0.073) | 0.91 | 0.94 | 0.97 | 0.94 (±0.018) |
| 5.6% | 0.97 | 0.99 | 0.98 | 0.98 (±0.006) | 0.70 | 0.85 | 0.80 | 0.78 (±0.044) | 1.38 | 1.17 | 1.22 | 1.26 (±0.066) | 0.82 | 0.81 | 0.84 | 0.82 (±0.008) |
| 10% | 1.05 | 1.08 | 1.03 | 1.05 (±0.015) | 0.77 | 0.80 | 0.87 | 0.81 (±0.029) | 1.36 | 1.35 | 1.19 | 1.30 (±0.056) | 0.69 | 0.67 | 0.74 | 0.70 (±0.020) |
| **Endpoint 🡪** | **Viability/Arachidonic Acid** | | | | **Thymidine** | | | | **2’-Deoxycytidine** | | | | **CPD** | |  |  |
| **[EVP-0]** | **Rep 1** | **Rep 2** | **Rep 3** | **Mean (±SEM)** | **Rep 1** | **Rep 2** | **Rep 3** | **Mean (±SEM)** | **Rep 1** | **Rep 2** | **Rep 3** | **Mean (±SEM)** |  |  |  |  |
| 0.18% | 0.96 | 0.96 | 0.99 | 0.97 (±0.010) | 1.04 | 0.95 | 0.97 | 0.99 (±0.028) | 1.05 | N/A | 1.04 | 1.04 (±0.004) | 0.926 | |  |  |
| 0.32% | 1.04 | 1.03 | 1.05 | 1.04 (±0.007) | 1.10 | 1.07 | 1.08 | 1.09 (±0.008) | 1.01 | 1.03 | 1.05 | 1.03 (±0.013) | 0.817 | |  |  |
| 0.56% | 0.98 | 1.08 | 0.92 | 0.99 (±0.049) | 0.99 | 1.09 | 1.07 | 1.05 (±0.031) | 0.97 | 1.04 | 1.06 | 1.02 (±0.026) | 0.827 | |  |  |
| 1.0% | 0.93 | 0.95 | 1.01 | 0.96 (±0.023) | 1.08 | 1.04 | 1.00 | 1.04 (±0.021) | 0.95 | 1.03 | 1.07 | 1.02 (±0.035) | 0.818 | |  |  |
| 1.8% | 1.04 | 0.95 | 1.03 | 1.01 (±0.026) | 1.10 | 1.06 | 1.02 | 1.06 (±0.023) | 1.03 | 1.01 | 1.03 | 1.02 (±0.007) | 0.814 | |  |  |
| 3.2% | 1.04 | 0.96 | 1.01 | 1.00 (±0.023) | 1.09 | 1.12 | 0.99 | 1.07 (±0.038) | 0.98 | 0.98 | 1.00 | 0.99 (±0.007) | 0.879 | |  |  |
| 5.6% | 1.18 | 1.22 | 1.17 | 1.19 (±0.017) | 0.98 | 1.08 | 1.06 | 1.04 (±0.030) | 1.05 | 0.95 | 0.99 | 1.00 (±0.030) | 0.873 | |  |  |
| 10% | 1.52 | 1.61 | 1.40 | 1.51 (±0.063) | 1.21 | 1.18 | 1.19 | 1.19 (±0.010) | 0.94 | 1.03 | 0.90 | 0.96 (±0.037) | 0.937 | |  |  |
| Values have been normalized to internal standards and reference controls  **CPD**: Combined Prediction Distance. **N/A**: Outlier value that was removed from analysis (determined by Grubbs’ test). | | | | | | | | | | | | | | | | |

| **Supplemental Table S20: Individual and Mean Endpoint Values for EVP-FB** | | | | | | | | | | | | | | | | |
| --- | --- | --- | --- | --- | --- | --- | --- | --- | --- | --- | --- | --- | --- | --- | --- | --- |
| **Endpoint 🡪** | **Cell Viability** | | | | **Lactic Acid** | | | | **Viability/Lactic Acid** | | | | **Arachidonic Acid** | | | |
| **[EVP-FB]** | **Rep 1** | **Rep 2** | **Rep 3** | **Mean (±SEM)** | **Rep 1** | **Rep 2** | **Rep 3** | **Mean (±SEM)** | **Rep 1** | **Rep 2** | **Rep 3** | **Mean (±SEM)** | **Rep 1** | **Rep 2** | **Rep 3** | **Mean (±SEM)** |
| 0.18% | 1.06 | 0.89 | 0.92 | 0.96 (±0.053) | 1.23 | 1.20 | 1.08 | 1.17 (±0.045) | 0.87 | 0.74 | 0.85 | 0.82 (±0.039) | 1.04 | 1.08 | 1.05 | 1.06 (±0.011) |
| 0.32% | 0.95 | 0.90 | 1.01 | 0.95 (±0.030) | 1.09 | 0.99 | 1.13 | 1.07 (±0.042) | 0.87 | 0.91 | 0.89 | 0.89 (±0.012) | 1.04 | 1.03 | 1.07 | 1.05 (±0.011) |
| 0.56% | 0.99 | 1.05 | 0.92 | 0.98 (±0.037) | 1.08 | 1.08 | 1.13 | 1.10 (±0.017) | 0.91 | 0.97 | 0.81 | 0.90 (±0.047) | 1.05 | 1.06 | 1.07 | 1.06 (±0.006) |
| 1.0% | 1.07 | 0.98 | 0.94 | 1.00 (±0.039) | 1.00 | 0.97 | 1.14 | 1.04 (±0.054) | 1.07 | 1.01 | 0.82 | 0.97 (±0.074) | 1.05 | 1.04 | 1.05 | 1.05 (±0.003) |
| 1.8% | 1.03 | N/A | 1.03 | 1.03 (±0.001) | 1.03 | N/A | 1.00 | 1.01 (±0.015) | 1.01 | N/A | 1.03 | 1.02 (±0.014) | 1.03 | 0.95 | 1.00 | 0.99 (±0.025) |
| 3.2% | 1.00 | 0.98 | 0.99 | 0.99 (±0.006) | 1.01 | 1.03 | 1.02 | 1.02 (±0.007) | 1.00 | 0.95 | 0.97 | 0.97 (±0.013) | 0.97 | 0.96 | 0.95 | 0.96 (±0.008) |
| 5.6% | 0.96 | 1.06 | 0.95 | 0.99 (±0.035) | 0.83 | 0.81 | 0.89 | 0.84 (±0.026) | 1.16 | 1.31 | 1.06 | 1.18 (±0.073) | 0.88 | 0.86 | 0.85 | 0.87 (±0.008) |
| 10% | 1.08 | 0.97 | 0.95 | 1.00 (±0.042) | 0.80 | 0.96 | 0.95 | 0.90 (±0.050) | 1.35 | 1.01 | 1.00 | 1.12 (±0.116) | 0.77 | 0.81 | 0.76 | 0.78 (±0.015) |
| **Endpoint 🡪** | **Viability/Arachidonic Acid** | | | | **Thymidine** | | | | **2’-Deoxycytidine** | | | | **CPD** | |  |  |
| **[EVP-FB]** | **Rep 1** | **Rep 2** | **Rep 3** | **Mean (±SEM)** | **Rep 1** | **Rep 2** | **Rep 3** | **Mean (±SEM)** | **Rep 1** | **Rep 2** | **Rep 3** | **Mean (±SEM)** |  |  |  |  |
| 0.18% | 1.02 | 0.83 | 0.88 | 0.91 (±0.058) | 1.06 | 1.06 | 1.05 | 1.06 (±0.002) | 1.01 | 0.99 | 1.05 | 1.02 (±0.020) | 0.805 | |  |  |
| 0.32% | 0.91 | 0.87 | 0.94 | 0.91 (±0.019) | 1.02 | 1.08 | 1.05 | 1.05 (±0.018) | 0.99 | 0.99 | 1.00 | 0.99 (±0.004) | 0.876 | |  |  |
| 0.56% | 0.94 | 0.98 | 0.86 | 0.93 (±0.036) | 0.92 | 1.08 | 1.06 | 1.02 (±0.051) | 1.07 | 1.01 | 1.01 | 1.03 (±0.020) | 0.805 | |  |  |
| 1.0% | 1.02 | 0.94 | 0.89 | 0.95 (±0.037) | 1.04 | 1.03 | 1.12 | 1.06 (±0.031) | 1.00 | 0.90 | 0.97 | 0.96 (±0.029) | 0.891 | |  |  |
| 1.8% | 1.00 | N/A | 1.03 | 1.02 (±0.014) | 1.05 | 1.14 | 1.18 | 1.12 (±0.040) | N/A | 1.10 | 1.07 | 1.09 (±0.018) | 0.861 | |  |  |
| 3.2% | 1.03 | 1.03 | 1.04 | 1.03 (±0.005) | 1.15 | 1.07 | 1.14 | 1.12 (±0.026) | 1.05 | 1.00 | 1.09 | 1.05 (±0.026) | 0.833 | |  |  |
| 5.6% | 1.09 | 1.22 | 1.11 | 1.14 (±0.041) | 1.09 | 1.05 | 1.06 | 1.07 (±0.013) | 1.04 | 1.12 | 1.01 | 1.06 (±0.031) | 0.826 | |  |  |
| 10% | 1.41 | 1.19 | 1.24 | 1.28 (±0.066) | 1.09 | 1.13 | 1.13 | 1.12 (±0.011) | 0.97 | 1.02 | 1.10 | 1.03 (±0.038) | 0.811 | |  |  |
| Values have been normalized to internal standards and reference controls  **CPD**: Combined Prediction Distance. **N/A**: Outlier value that was removed from analysis (determined by Grubbs’ test). | | | | | | | | | | | | | | |  |  |

| **Supplemental Table S21: Individual and Mean Endpoint Values for EVP-NS** | | | | | | | | | | | | | | | | |
| --- | --- | --- | --- | --- | --- | --- | --- | --- | --- | --- | --- | --- | --- | --- | --- | --- |
| **Endpoint 🡪** | **Cell Viability** | | | | **Lactic Acid** | | | | **Viability/Lactic Acid** | | | | **Arachidonic Acid** | | | |
| **[EVP-NS]** | **Rep 1** | **Rep 2** | **Rep 3** | **Mean (±SEM)** | **Rep 1** | **Rep 2** | **Rep 3** | **Mean (±SEM)** | **Rep 1** | **Rep 2** | **Rep 3** | **Mean (±SEM)** | **Rep 1** | **Rep 2** | **Rep 3** | **Mean (±SEM)** |
| 0.18% | 1.07 | 1.00 | 0.96 | 1.01 (±0.031) | 1.02 | 1.09 | 1.14 | 1.08 (±0.035) | 1.04 | 0.92 | 0.84 | 0.93 (±0.060) | 1.11 | 1.09 | 1.10 | 1.10 (±0.006) |
| 0.32% | 1.07 | 0.97 | 1.02 | 1.02 (±0.029) | 1.03 | 1.07 | 1.13 | 1.08 (±0.029) | 1.04 | 0.91 | 0.90 | 0.95 (±0.044) | 1.08 | 1.18 | 1.11 | 1.12 (±0.030) |
| 0.56% | 1.00 | 0.97 | 1.07 | 1.01 (±0.029) | 1.00 | 0.98 | 0.91 | 0.97 (±0.026) | 1.00 | 0.99 | 1.17 | 1.05 (±0.059) | 1.11 | 1.12 | 1.18 | 1.14 (±0.023) |
| 1.0% | 1.03 | 1.03 | 1.05 | 1.04 (±0.008) | 0.94 | 1.05 | 0.95 | 0.98 (±0.034) | 1.10 | 0.98 | 1.11 | 1.07 (±0.041) | 1.09 | 1.10 | 1.12 | 1.10 (±0.012) |
| 1.8% | 0.98 | 0.97 | 1.01 | 0.98 (±0.013) | 0.95 | 0.96 | 0.92 | 0.95 (±0.011) | 1.02 | 1.01 | 1.09 | 1.04 (±0.026) | 1.10 | 1.23 | 1.14 | 1.15 (±0.037) |
| 3.2% | 0.97 | 0.92 | 1.01 | 0.96 (±0.025) | 0.83 | 0.97 | 0.89 | 0.90 (±0.039) | 1.16 | 0.95 | 1.13 | 1.08 (±0.065) | 1.05 | 1.03 | 1.10 | 1.06 (±0.022) |
| 5.6% | 0.99 | 1.00 | 1.07 | 1.02 (±0.024) | 0.86 | 0.75 | 0.76 | 0.79 (±0.036) | 1.15 | 1.33 | 1.40 | 1.29 (±0.075) | 0.98 | 0.97 | 1.03 | 0.99 (±0.017) |
| 10% | 0.98 | 1.07 | 1.05 | 1.03 (±0.028) | 0.68 | N/A | 0.77 | 0.73 (±0.045) | 1.43 | N/A | 1.35 | 1.39 (±0.037) | 0.90 | N/A | 0.86 | 0.88 (±0.021) |
| **Endpoint 🡪** | **Viability/Arachidonic Acid** | | | | **Thymidine** | | | | **2’-Deoxycytidine** | | | | **CPD** | |  |  |
| **[EVP-NS]** | **Rep 1** | **Rep 2** | **Rep 3** | **Mean (±SEM)** | **Rep 1** | **Rep 2** | **Rep 3** | **Mean (±SEM)** | **Rep 1** | **Rep 2** | **Rep 3** | **Mean (±SEM)** |  |  |  |  |
| 0.18% | 0.96 | 0.92 | 0.87 | 0.92 (±0.027) | 1.11 | 1.00 | 1.01 | 1.04 (±0.035) | 1.15 | 1.10 | N/A | 1.12 (±0.028) | 0.893 | |  |  |
| 0.32% | 0.99 | 0.82 | 0.92 | 0.91 (±0.049) | 1.02 | 1.05 | 1.16 | 1.08 (±0.043) | 0.86 | 0.93 | 1.26 | 1.02 (±0.122) | 0.926 | |  |  |
| 0.56% | 0.90 | 0.87 | 0.90 | 0.89 (±0.010) | 1.10 | 1.05 | 0.98 | 1.04 (±0.037) | 0.96 | 0.95 | 1.08 | 1.00 (±0.040) | 0.900 | |  |  |
| 1.0% | 0.95 | 0.94 | 0.94 | 0.94 (±0.005) | 1.01 | 1.01 | 1.01 | 1.01 (±0.001) | 1.11 | 1.04 | 0.94 | 1.03 (±0.051) | 0.824 | |  |  |
| 1.8% | 0.89 | 0.79 | 0.89 | 0.85 (±0.033) | 1.05 | 1.04 | 1.02 | 1.04 (±0.009) | 1.07 | 1.04 | 1.03 | 1.04 (±0.013) | 0.822 | |  |  |
| 3.2% | 0.92 | 0.89 | 0.91 | 0.91 (±0.009) | 1.06 | 1.04 | 1.03 | 1.04 (±0.009) | 0.96 | 0.85 | 1.01 | 0.94 (±0.048) | 0.905 | |  |  |
| 5.6% | 1.02 | 1.03 | 1.04 | 1.03 (±0.007) | 1.10 | 1.08 | 0.98 | 1.06 (±0.037) | 1.10 | N/A | 1.08 | 1.09 (±0.006) | 0.926 | |  |  |
| 10% | 1.08 | N/A | 1.22 | 1.15 (±0.068) | 1.21 | N/A | 1.13 | 1.17 (±0.041) | 0.88 | N/A | 0.99 | 0.94 (±0.052) | 0.965 | |  |  |
| Values have been normalized to internal standards and reference controls  **CPD**: Combined Prediction Distance. **N/A**: Outlier value that was removed from analysis (determined by Grubbs’ test). | | | | | | | | | | | | | | |  |  |

| **Supplemental Table S22: Individual and Mean Endpoint Values for cHTP** | | | | | | | | | | | | | | | | |
| --- | --- | --- | --- | --- | --- | --- | --- | --- | --- | --- | --- | --- | --- | --- | --- | --- |
| **Endpoint 🡪** | **Cell Viability** | | | | **Lactic Acid** | | | | **Viability/Lactic Acid** | | | | **Arachidonic Acid** | | | |
| **[cTHP]** | **Rep 1** | **Rep 2** | **Rep 3** | **Mean (±SEM)** | **Rep 1** | **Rep 2** | **Rep 3** | **Mean (±SEM)** | **Rep 1** | **Rep 2** | **Rep 3** | **Mean (±SEM)** | **Rep 1** | **Rep 2** | **Rep 3** | **Mean (±SEM)** |
| 0.18% | 0.99 | 0.93 | 0.97 | 0.96 (±0.020) | 1.05 | 1.14 | 1.05 | 1.08 (±0.031) | 0.95 | 0.81 | 0.92 | 0.89 (±0.042) | 1.20 | 1.16 | 1.12 | 1.16 (±0.023) |
| 0.32% | 1.07 | 0.98 | 1.00 | 1.02 (±0.026) | 0.99 | 1.10 | 1.15 | 1.08 (±0.047) | 1.07 | 0.89 | 0.86 | 0.94 (±0.066) | 1.14 | 1.16 | 1.15 | 1.15 (±0.004) |
| 0.56% | 1.04 | 1.05 | 0.99 | 1.03 (±0.020) | 0.98 | 1.03 | 1.02 | 1.01 (±0.017) | 1.07 | 1.02 | 0.97 | 1.02 (±0.029) | 1.20 | 1.17 | 1.27 | 1.21 (±0.030) |
| 1.0% | 1.06 | 1.09 | 0.97 | 1.04 (±0.038) | 0.91 | 0.87 | 0.96 | 0.91 (±0.025) | 1.18 | 1.26 | 1.01 | 1.15 (±0.072) | 1.16 | 1.18 | 1.14 | 1.16 (±0.010) |
| 1.8% | 1.07 | 0.94 | 0.97 | 1.00 (±0.040) | 0.83 | 0.83 | 0.74 | 0.80 (±0.029) | 1.30 | 1.14 | 1.31 | 1.25 (±0.055) | 1.16 | 1.19 | 1.20 | 1.18 (±0.012) |
| 3.2% | 1.10 | 1.04 | 1.03 | 1.06 (±0.021) | 0.71 | 0.72 | 0.70 | 0.71 (±0.007) | 1.55 | 1.44 | 1.48 | 1.49 (±0.032) | 1.21 | 1.15 | 1.11 | 1.16 (±0.030) |
| 5.6% | 1.07 | 1.03 | 1.08 | 1.06 (±0.015) | 0.60 | 0.61 | N/A | 0.60 (±0.005) | 1.79 | 1.70 | N/A | 1.74 (±0.046) | 1.02 | 1.04 | 1.01 | 1.02 (±0.006) |
| 10% | 0.81 | 0.75 | 0.65 | 0.73 (±0.046) | 0.99 | 1.55 | 2.16 | 1.57 (±0.337) | 0.81 | 0.48 | 0.30 | 0.53 (±0.149) | 1.20 | 1.39 | 1.54 | 1.38 (±0.099) |
| **Endpoint 🡪** | **Viability/Arachidonic Acid** | | | | **Thymidine** | | | | **2’-Deoxycytidine** | | | | **CPD** | |  |  |
| **[cHTP]** | **Rep 1** | **Rep 2** | **Rep 3** | **Mean (±SEM)** | **Rep 1** | **Rep 2** | **Rep 3** | **Mean (±SEM)** | **Rep 1** | **Rep 2** | **Rep 3** | **Mean (±SEM)** |  |  |  |  |
| 0.18% | 0.83 | 0.80 | 0.87 | 0.83 (±0.020) | 1.07 | 1.03 | 1.23 | 1.11 (±0.060) | 0.94 | 1.04 | 1.18 | 1.05 (±0.071) | 0.825 | |  |  |
| 0.32% | 0.93 | 0.85 | 0.87 | 0.88 (±0.026) | 1.10 | 1.14 | 0.94 | 1.06 (±0.059) | 1.11 | 1.10 | 0.96 | 1.06 (±0.049) | 0.873 | |  |  |
| 0.56% | 0.87 | 0.90 | 0.78 | 0.85 (±0.037) | 1.04 | 1.03 | 1.11 | 1.06 (±0.025) | 1.19 | 0.88 | 0.94 | 1.00 (±0.095) | 0.925 | |  |  |
| 1.0% | 0.92 | 0.93 | 0.85 | 0.90 (±0.026) | 1.01 | 1.04 | 1.09 | 1.05 (±0.025) | 0.95 | 1.00 | 1.13 | 1.03 (±0.054) | 0.868 | |  |  |
| 1.8% | 0.93 | 0.79 | 0.81 | 0.85 (±0.042) | 1.04 | 1.01 | 1.07 | 1.04 (±0.018) | 1.05 | 0.93 | 0.92 | 0.97 (±0.041) | 0.927 | |  |  |
| 3.2% | 0.91 | 0.90 | 0.93 | 0.91 (±0.010) | 1.06 | 0.99 | 1.03 | 1.03 (±0.020) | 1.15 | 1.08 | 1.17 | 1.14 (±0.028) | 1.026 | |  |  |
| 5.6% | 1.04 | 0.99 | 1.06 | 1.03 (±0.021) | 1.04 | 1.02 | 0.98 | 1.01 (±0.017) | 1.03 | 1.05 | 0.80 | 0.96 (±0.081) | 1.210 | |  |  |
| 10% | 0.67 | 0.54 | 0.42 | 0.54 (±0.073) | 0.69 | 0.63 | 0.27 | 0.53 (±0.132) | 0.62 | 0.52 | 0.48 | 0.54 (±0.040) | 1.658 | |  |  |
| Values have been normalized to internal standards and reference controls  **CPD**: Combined Prediction Distance. **N/A**: Outlier value that was removed from analysis (determined by Grubbs’ test). | | | | | | | | | | | | | | |  |  |

| **Supplemental Table S23: Individual and Mean Endpoint Values for pHTP 1** | | | | | | | | | | | | | | | | |
| --- | --- | --- | --- | --- | --- | --- | --- | --- | --- | --- | --- | --- | --- | --- | --- | --- |
| **Endpoint 🡪** | **Cell Viability** | | | | **Lactic Acid** | | | | **Viability/Lactic Acid** | | | | **Arachidonic Acid** | | | |
| **[pHTP 1]** | **Rep 1** | **Rep 2** | **Rep 3** | **Mean (±SEM)** | **Rep 1** | **Rep 2** | **Rep 3** | **Mean (±SEM)** | **Rep 1** | **Rep 2** | **Rep 3** | **Mean (±SEM)** | **Rep 1** | **Rep 2** | **Rep 3** | **Mean (±SEM)** |
| 0.18% | 0.95 | 0.94 | 1.01 | 0.97 (±0.02) | 0.96 | 0.95 | 0.93 | 0.95 (±0.01) | 0.99 | 0.98 | 1.09 | 1.02 (±0.03) | 0.99 | 0.96 | 0.97 | 0.97 (±0.01) |
| 0.32% | 0.97 | 0.91 | 1.00 | 0.96 (±0.03) | 0.97 | 0.96 | 1.13 | 1.02 (±0.05) | 1.00 | 0.94 | 0.88 | 0.94 (±0.04) | N/A | 1.04 | 1.04 | 1.04 (±0.00) |
| 0.56% | 0.90 | 0.95 | 1.02 | 0.96 (±0.04) | 1.09 | 0.96 | 0.92 | 0.99 (±0.05) | 0.83 | 0.99 | 1.11 | 0.98 (±0.08) | 0.99 | 1.00 | 1.03 | 1.01 (±0.01) |
| 1.0% | 0.99 | 1.01 | 1.02 | 1.01 (±0.01) | 0.88 | 0.96 | 0.84 | 0.90 (±0.04) | 1.12 | 1.05 | 1.22 | 1.13 (±0.05) | 1.04 | 1.03 | 1.04 | 1.04 (±0.01) |
| 1.8% | 0.98 | 0.93 | 1.00 | 0.97 (±0.02) | 0.84 | 0.89 | 0.85 | 0.86 (±0.01) | 1.16 | 1.05 | 1.18 | 1.13 (±0.04) | 1.00 | 0.98 | 1.03 | 1.00 (±0.01) |
| 3.2% | 0.97 | 0.95 | 0.97 | 0.96 (±0.01) | 0.81 | 0.78 | 0.78 | 0.79 (±0.01) | 1.19 | 1.21 | 1.25 | 1.22 (±0.02) | 0.98 | 1.01 | 1.08 | 1.03 (±0.03) |
| 5.6% | 0.98 | 0.96 | 1.01 | 0.98 (±0.01) | 0.69 | 0.74 | 0.83 | 0.75 (±0.04) | 1.43 | 1.31 | 1.22 | 1.32 (±0.06) | N/A | 1.08 | 1.08 | 1.08 (±0.00) |
| 10% | 0.92 | 0.89 | 0.93 | 0.91 (±0.01) | 0.51 | 0.53 | 0.44 | 0.50 (±0.03) | 1.78 | 1.67 | 2.11 | 1.85 (±0.13) | 0.92 | 0.96 | 0.95 | 0.94 (±0.01) |
| **Endpoint 🡪** | **Viability/Arachidonic Acid** | | | | **Thymidine** | | | | **2’-Deoxycytidine** | | | | **CPD** | |  |  |
| **[pHTP 1]** | **Rep 1** | **Rep 2** | **Rep 3** | **Mean (±SEM)** | **Rep 1** | **Rep 2** | **Rep 3** | **Mean (±SEM)** | **Rep 1** | **Rep 2** | **Rep 3** | **Mean (±SEM)** |  |  |  |  |
| 0.18% | 0.97 | 0.98 | 1.04 | 0.99 (±0.02) | 1.00 | N/A | 1.07 | 1.04 (±0.04) | 1.09 | 1.02 | 1.10 | 1.07 (±0.03) | 0.867 | |  |  |
| 0.32% | N/A | 0.87 | 0.96 | 0.91 (±0.04) | 0.90 | 1.02 | 1.06 | 0.99 (±0.05) | 1.00 | 1.11 | 1.00 | 1.03 (±0.04) | 0.868 | |  |  |
| 0.56% | 0.90 | 0.95 | 0.99 | 0.95 (±0.02) | 1.17 | 1.04 | 0.98 | 1.06 (±0.06) | 1.14 | 1.12 | 0.97 | 1.08 (±0.05) | 0.886 | |  |  |
| 1.0% | 0.95 | 0.99 | 0.98 | 0.97 (±0.01) | 0.90 | 1.00 | 0.69 | 0.86 (±0.09) | 1.16 | 1.05 | 1.06 | 1.09 (±0.04) | 0.994 | |  |  |
| 1.8% | 0.98 | 0.95 | 0.97 | 0.97 (±0.01) | 0.93 | 1.01 | 1.01 | 0.98 (±0.03) | 1.04 | 0.98 | 1.03 | 1.02 (±0.02) | 0.817 | |  |  |
| 3.2% | 0.98 | 0.94 | 0.90 | 0.94 (±0.02) | 1.11 | 1.05 | 1.04 | 1.07 (±0.02) | 1.14 | 1.02 | 0.93 | 1.03 (±0.06) | 0.839 | |  |  |
| 5.6% | N/A | 0.89 | 0.93 | 0.91 (±0.02) | N/A | 0.99 | 0.97 | 0.98 (±0.01) | 1.01 | N/A | 1.01 | 1.01 (±0.00) | 0.914 | |  |  |
| 10% | 1.00 | 0.92 | 0.98 | 0.97 (±0.02) | 0.92 | 0.81 | 0.82 | 0.85 (±0.04) | 0.93 | 0.88 | N/A | 0.91 (±0.03) | 1.236 | |  |  |
| Values have been normalized to internal standards and reference controls  **CPD**: Combined Prediction Distance. **N/A**: Outlier value that was removed from analysis (determined by Grubbs’ test). | | | | | | | | | | | | | | | | |

| **Supplemental Table S24: Individual and Mean Endpoint Values for pTHP 2** | | | | | | | | | | | | | | | | |
| --- | --- | --- | --- | --- | --- | --- | --- | --- | --- | --- | --- | --- | --- | --- | --- | --- |
| **Endpoint 🡪** | **Cell Viability** | | | | **Lactic Acid** | | | | **Viability/Lactic Acid** | | | | **Arachidonic Acid** | | | |
| **[pHTP 2]** | **Rep 1** | **Rep 2** | **Rep 3** | **Mean (±SEM)** | **Rep 1** | **Rep 2** | **Rep 3** | **Mean (±SEM)** | **Rep 1** | **Rep 2** | **Rep 3** | **Mean (±SEM)** | **Rep 1** | **Rep 2** | **Rep 3** | **Mean (±SEM)** |
| 0.18% | 0.94 | 0.95 | 1.00 | 0.96 (±0.02) | 1.01 | 1.00 | 1.00 | 1.01 (±0.00) | 0.93 | 0.94 | 1.00 | 0.96 (±0.02) | 0.98 | 0.97 | 0.97 | 0.97 (±0.00) |
| 0.32% | 0.94 | 0.97 | 1.00 | 0.97 (±0.02) | 1.01 | 0.96 | 1.06 | 1.01 (±0.03) | 0.93 | 1.02 | 0.95 | 0.97 (±0.03) | 0.99 | 0.96 | 0.99 | 0.98 (±0.01) |
| 0.56% | 0.94 | 1.00 | 1.04 | 0.99 (±0.03) | 0.89 | 0.98 | 0.89 | 0.92 (±0.03) | 1.06 | 1.02 | 1.17 | 1.08 (±0.04) | 0.98 | 1.00 | 1.00 | 0.99 (±0.01) |
| 1.0% | 0.95 | 0.99 | 1.04 | 0.99 (±0.02) | 1.02 | 0.84 | 0.88 | 0.91 (±0.05) | 0.94 | 1.17 | 1.18 | 1.10 (±0.08) | 1.09 | 1.12 | 1.03 | 1.08 (±0.03) |
| 1.8% | 0.94 | 0.95 | 1.01 | 0.97 (±0.02) | 0.85 | 0.90 | 0.77 | 0.84 (±0.04) | 1.11 | 1.06 | 1.31 | 1.16 (±0.07) | 1.04 | 1.11 | 1.05 | 1.07 (±0.02) |
| 3.2% | 0.98 | 0.98 | 1.00 | 0.99 (±0.00) | 0.77 | 0.90 | 0.69 | 0.79 (±0.06) | 1.27 | 1.09 | 1.44 | 1.26 (±0.10) | 1.07 | 1.16 | 1.01 | 1.08 (±0.04) |
| 5.6% | 0.99 | 1.00 | 1.02 | 1.01 (±0.01) | 0.93 | 0.78 | 1.03 | 0.91 (±0.07) | 1.06 | 1.29 | 1.00 | 1.12 (±0.09) | 1.03 | 1.11 | 1.09 | 1.08 (±0.03) |
| 10% | 1.02 | N/A | 1.02 | 1.02 (±0.00) | 0.55 | 0.61 | 0.57 | 0.58 (±0.02) | 1.85 | N/A | 1.78 | 1.81 (±0.03) | 1.07 | 1.11 | 1.08 | 1.09 (±0.01) |
| **Endpoint 🡪** | **Viability/Arachidonic Acid** | | | | **Thymidine** | | | | **2’-Deoxycytidine** | | | | **CPD** | |  |  |
| **[pHTP 2]** | **Rep 1** | **Rep 2** | **Rep 3** | **Mean (±SEM)** | **Rep 1** | **Rep 2** | **Rep 3** | **Mean (±SEM)** | **Rep 1** | **Rep 2** | **Rep 3** | **Mean (±SEM)** |  |  |  |  |
| 0.18% | 0.96 | 0.98 | 1.03 | 0.99 (±0.02) | 0.98 | 1.12 | 0.99 | 1.03 (±0.05) | 1.05 | N/A | 1.11 | 1.08 (±0.03) | 0.908 | |  |  |
| 0.32% | 0.95 | 1.02 | 1.01 | 0.99 (±0.02) | 1.18 | 0.96 | 1.16 | 1.10 (±0.07) | 0.92 | 0.92 | 0.96 | 0.93 (±0.02) | 0.942 | |  |  |
| 0.56% | 0.96 | 1.00 | 1.04 | 1.00 (±0.02) | 1.14 | 1.30 | 1.02 | 1.16 (±0.08) | 0.98 | 1.07 | 1.09 | 1.05 (±0.03) | 0.845 | |  |  |
| 1.0% | 0.88 | 0.88 | 1.00 | 0.92 (±0.04) | 1.07 | 1.16 | 1.17 | 1.13 (±0.03) | 0.88 | 0.90 | 1.02 | 0.93 (±0.04) | 0.964 | |  |  |
| 1.8% | 0.90 | 0.86 | 0.96 | 0.91 (±0.03) | 1.04 | 1.17 | 1.01 | 1.07 (±0.05) | 0.95 | 1.06 | 0.97 | 0.99 (±0.03) | 0.889 | |  |  |
| 3.2% | 0.92 | 0.85 | 0.98 | 0.92 (±0.04) | 1.12 | 1.01 | 1.13 | 1.09 (±0.04) | 1.04 | 1.02 | 0.94 | 1.00 (±0.03) | 0.879 | |  |  |
| 5.6% | 0.97 | 0.90 | 0.94 | 0.94 (±0.02) | 1.22 | 1.11 | 1.22 | 1.18 (±0.03) | 0.98 | 0.93 | 0.96 | 0.95 (±0.01) | 0.905 | |  |  |
| 10% | 0.95 | N/A | 0.95 | 0.95 (±0.00) | 1.05 | 0.98 | 0.53 | 0.85 (±0.16) | 1.14 | 0.86 | 0.95 | 0.98 (±0.08) | 1.259 | |  |  |
| Values have been normalized to internal standards and reference controls  **CPD**: Combined Prediction Distance. **N/A**: Outlier value that was removed from analysis (determined by Grubbs’ test). | | | | | | | | | | | | | | | | |
